# The Neurogenic Potential of Astrocytes Is Regulated by Inflammatory Signals

**DOI:** 10.1007/s12035-015-9296-x

**Published:** 2015-07-04

**Authors:** Alessandro Michelucci, Angela Bithell, Matthew J. Burney, Caroline E. Johnston, Kee-Yew Wong, Siaw-Wei Teng, Jyaysi Desai, Nigel Gumbleton, Gregory Anderson, Lawrence W. Stanton, Brenda P. Williams, Noel J. Buckley

**Affiliations:** 1 Institute of Psychiatry, Centre for the Cellular Basis of Behaviour, The James Black Centre, King’s College London, 125 Coldharbour Lane, London, SE5 9NU UK; 2 Luxembourg Centre for Systems Biomedicine, University of Luxembourg, Campus Belval, 7, Avenue des Hauts-Fourneaux, L-4362 Esch-Belval, Luxembourg; 3 School of Pharmacy, Department of Pharmacology, The Hopkins Building, University of Reading, Whiteknights, Reading, RG6 6AP UK; 4 Genome Institute of Singapore, 60 Biopolis Street, #02-01, Genome Building, Singapore, 138672 Singapore; 5 Department of Psychiatry, Warneford Hospital, University of Oxford, Warneford Lane, Oxford, OX3 7JX UK

**Keywords:** Inflammation, Astrocytes, Neural stem cells, Noggin, NFκB, Epigenetic

## Abstract

Although the adult brain contains neural stem cells (NSCs) that generate new neurons throughout life, these astrocyte-like populations are restricted to two discrete niches. Despite their terminally differentiated phenotype, adult parenchymal astrocytes can re-acquire NSC-like characteristics following injury, and as such, these ‘reactive’ astrocytes offer an alternative source of cells for central nervous system (CNS) repair following injury or disease. At present, the mechanisms that regulate the potential of different types of astrocytes are poorly understood. We used in vitro and ex vivo astrocytes to identify candidate pathways important for regulation of astrocyte potential. Using in vitro neural progenitor cell (NPC)-derived astrocytes, we found that exposure of more lineage-restricted astrocytes to either tumor necrosis factor alpha (TNF-α) (via nuclear factor-κB (NFκB)) or the bone morphogenetic protein (BMP) inhibitor, noggin, led to re-acquisition of NPC properties accompanied by transcriptomic and epigenetic changes consistent with a more neurogenic, NPC-like state. Comparative analyses of microarray data from in vitro-derived and ex vivo postnatal parenchymal astrocytes identified several common pathways and upstream regulators associated with inflammation (including transforming growth factor (TGF)-β1 and peroxisome proliferator-activated receptor gamma (PPARγ)) and cell cycle control (including TP53) as candidate regulators of astrocyte phenotype and potential. We propose that inflammatory signalling may control the normal, progressive restriction in potential of differentiating astrocytes as well as under reactive conditions and represent future targets for therapies to harness the latent neurogenic capacity of parenchymal astrocytes.

**Electronic supplementary material:**

The online version of this article (doi:10.1007/s12035-015-9296-x) contains supplementary material, which is available to authorized users.

## Introduction

Astrocytes were historically seen as support cells in the central nervous system (CNS) and in fact discharge multiple functions including regulation of energy metabolism, calcium signalling, synaptic transmission and mediating inflammatory responses. This view has been expanded by the recognition that astrocytes are also highly heterogeneous [1–3]. Interestingly, neurogenic adult neural stem cells (aNSCs) that reside in two niches (the subventricular zone (SVZ) of the lateral ventricles and the subgranular zone (SGZ) of the dentate gyrus [4, 5]) have an astrocyte-like phenotype [6]. Even more intriguingly, there is a growing body of evidence that some parenchymal astrocytes have a latent neurogenic capacity. These observations underlie the need to identify regulatory pathways that govern the ability of an astrocyte to express NSC properties.

Early evidence for the neurogenic potential of parenchymal astrocytes came from observations that immature astrocytes from neonatal mouse adopt a radial glia-like phenotype when cultured with embryonic day 14 (E14) cortical cells [7], whilst astrocytes from embryonic or neonatal brain can form multipotent neurospheres [8]. Furthermore, forced expression of neurogenic transcription factors including Mash1, Ngn2 or Dlx2 is capable of converting postnatal parenchymal astrocytes to functional neurons [9–12]. Collectively, these studies show that immature parenchymal astrocytes have a latent neurogenic potential that can be realised by manipulation of intrinsic transcriptional programmes or the cellular milieu. Importantly, astrocytes from more mature brain after postnatal day 10 (P10) are not capable of generating neurospheres, indicating that the ability to dedifferentiate is a unique property of immature astrocytes. Intriguingly, under inflammatory conditions or following injury, mature parenchymal astrocytes can become reactive and re-acquire more immature or neural progenitor cell (NPC)-like properties [6, 13, 14]. Furthermore, reactive astrocytes isolated from adult cortex can give rise to multipotent neurospheres [15]. These observations underlie the relevance of understanding the latent neurogenic capacity of astrocytes for regenerative medicine strategies designed to recruit astrocytes into repair of damaged brain. Reactive astrocytes are, by definition, present at the site of injury and therefore offer an advantage over niche aNSCs that may reside far from the injury site. Although these studies clearly demonstrate the latent neurogenic capacity of some parenchymal astrocytes, the signalling pathways and mechanisms that regulate the reprogramming of astrocytes to NPC-like states or directly to neurons remain largely unknown [16].

The drive to identify genes for ‘stemness’ was initially led by transcriptome studies [17, 18], but recently, the idea has emerged that epigenetic signatures (including post-translational modification to histones and DNA methylation) can provide an indicator of cellular potential [19–24]. Indeed, epigenetic reprogramming is key to the generation of induced pluripotent stem cells (iPSCs). Whilst we are beginning to unravel the role of epigenetics in NSCs and neuronal differentiation, understanding of its contribution to maturation and reactivation of astrocytes is poor.

Our aim was to explore the transcriptome and epigenetic profile of different astrocyte populations to identify molecular signatures of astrocyte plasticity. To do this, we used homogeneous populations of NPC-derived astrocytes that show a differential ability to revert to an NPC-like state. We identified two factors: the pro-inflammatory cytokine, tumor necrosis factor alpha (TNF-α), known to play a role in reactive gliosis and NPC proliferation [25–27] and the BMP antagonist, noggin, as key regulators that govern reprogramming of astrocytes to NPCs. We also show that changes in epigenetic profiles accompany changes in cellular potential. Importantly, by comparing in vitro and ex vivo astrocyte transcriptomes, we provide evidence that there are likely to be common pathways and regulators responsible for astrocyte identity and potential in normal and injured brain that include pro- and anti-inflammatory signalling. Further, we find that astrocyte potential is also reflected in their intrinsic epigenetic signatures. These data add to a growing repository that aids identification of regulatory pathways involved in maintenance or re-acquisition of neurogenic NPC potential that may allow recruitment of parenchymal astrocytes in repair strategies for treatment of brain injury and degeneration.

## Materials and Methods

### Mice

All UK animal handling and procedures were performed according to the UK Animals (Scientific Procedures) Act, 1986 under Home Office licence. All animal procedures in Luxembourg were performed according to Federation of European Laboratory Animal Science Associations (FELASA) guidelines for the use of animals in research. Transgenic mice were genotyped using standard protocols and specific primers (Table S1).

### Cell Culture

#### Cell Line

The CTX12 cell line is a conditionally immortalised mouse NPC line derived from E12-5 mouse cortex that has been generated in-house at King’s College London by Dr. Bithell and Dr. Williams. Cells were grown on poly-d-lysine (PDL)/laminin-coated plastic (Sigma) in modified Sato’s medium (*modified Sato*’*s medium*: Dulbecco’s modified Eagle’s medium (DMEM)/F12 supplemented with 5.6 mg/ml glucose, 100 μg/ml bovine serum albumin (BSA), 16 μg/ml putrescine, 60 ng/ml progesterone, 400 ng/ml l-thyroxine, 300 ng/ml 3,3′,5-triiodothyronine, 5 μg/ml insulin, 5 μg/ml apo-transferrin, 5 ng/ml sodium selenite, 1× glutamine, 1× pen/strep) containing 10 ng/ml fibroblast growth factor 2 (FGF2), 20 ng/ml epidermal growth factor (EGF) and 100 nM 4-hydroxytamoxifen (4-OHT). Medium was changed every 2–3 days and cells passaged using trypsin-EDTA and trypsin inhibitor (Sigma). For astrocyte differentiation, CTX12 cells were plated at 0.5 × 10^5^ cells/cm^2^ and cultured in modified Sato’s medium with 10 % foetal bovine serum (FBS) or 20 ng/ml bone morphogenetic protein 4 (BMP4) (Peprotech and R&D Systems). Where indicated, cells were treated with additional factors: noggin (500 ng/ml), TNF-α (50 ng/ml) (Peprotech) and JSH-23 nuclear factor-κB (NFκB) Activation Inhibitor II (JSH-23, 10 μM, Santa Cruz).

#### Primary Cells

Cortices were isolated from P21 Swiss Webster and collected in calcium/magnesium-free Hank’s balanced salt solution (HBSS), trypsinised and DNAseI treated (50 μg/ml, Sigma) for 20 min at 37 °C and mechanically dissociated into a homogenous cell suspension [28]. Following washes and centrifugation, mixed glial cells were plated onto PDL (Sigma) in DMEM/F12 (Invitrogen) with 100 U/ml penicillin/100 mg/ml streptomycin (Sigma) and 10 % FBS (Biosera). Once confluent, cultures were shaken overnight at 180 rpm to remove microglia and oligodendrocytes and treated for 4–7 days with 20 μM cytosine arabinoside (AraC) to kill remaining dividing cells and obtain essentially pure astrocytes (>98 %), determined by immunofluorescence using anti-glial fibrillary acidic protein (GFAP) (Millipore) and anti-S100β (Dako) for astrocytes, anti-Iba1 (Biocare, microglia), anti-O4 (Sigma, oligodendrocytes) and TuJ1 (Covance, neurons).

### Preparation of Mouse Forebrain Cell Suspensions and Fluorescence-Activated Cell Sorting (FACS) of Astrocytes

Different developmental stages (P4, P10 or P21) of forebrains from *Aldh1l1*-*EGFP* (*Fthfd*) transgenic mice (GenSat/MMRRC) were collected in calcium/magnesium-free HBSS. Tissue was diced and papain digested at 33 °C for 90 min (20 U/ml, Sigma) in dissociation buffer [EBSS (Sigma), D(+)-glucose 22.5 mM, NaHCO_3_ 26 mM and DNaseI 125 U/ml with EDTA 0.5 mM and l-cysteine–HCl 1 mM (Sigma)] and washed 3× in dissociation buffer with BSA (1.0 mg/ml, Sigma) and trypsin inhibitor (1.0 mg/ml, Sigma) before mechanical dissociation through 5 ml and fire-polished Pasteur pipettes to a single cell suspension. Cells were pelleted, resuspended in cold phosphate-buffered saline (PBS) with DNaseI at 1 × 10^6^ cell/ml, passed through a 70-μm filter and 7-aminoactinomycin D (7-AAD, Sigma) added. FITC-positive/PE-Cy5-negative cells were sorted. FACS was performed using a FACSAria I SORP running FACSDiva6.3 software (BD Biosciences).

### Immunocytochemistry

Cells were fixed for 10 min in 4 % paraformaldehyde, permeabilised and incubated with primary antibodies in 1× PBS with 10 % normal serum. Primary antibodies used were anti-Olig2 (1:500; Millipore), anti-GFAP (1:400; Millipore), anti-Ki67 (1:1,000; Abcam), anti-Nestin (1:1,000; Abcam), anti-Sox2 (1:200; Santa Cruz), anti-O4 (Sigma, 1:200), anti-Iba1 (1:200, Biocare), TuJ1 (1:1,000, Covance), anti-GFP (1:2,000, Abcam), anti-bromodeoxyuridine (BrdU) (1:250, Abcam) and anti-NFκB-p65 (1:500; Abcam). Primary antibodies were visualised using specific AlexaFluor secondary antibodies (Molecular Probes), and nuclei were counterstained with 4′,6-diamidino-2-phenylindole (DAPI). Coverslips were mounted in Prolong Gold anti-fade mounting medium (Molecular Probes) and analysed using Zeiss AxioImager Z1 microscopes and AxioVision software.

#### BrdU Labelling

Cells were pulsed with BrdU (100 μM, Sigma) for 24 h, fixed with 4 % paraformaldehyde and treated with 2 N HCl for 45 min and 0.1 M borax (pH 8.5) for 15 min before processing for immunocytochemistry (above).

### RNA Isolation, Microarray Hybridisation and Data Analysis

Cells were pelleted and lysed with TRIzol reagent (Invitrogen). Total RNA was further purified using an RNeasy Mini Kit (Qiagen). Biological replicates were prepared for microarrays using an Illumina TotalPrep RNA Amplification Kit (Ambion) and amplified RNAs hybridised on Sentrix® Mouse Ref-8 Expression BeadChips (Illumina), washed and scanned with Illumina BeadStation according to the Illumina protocols. Raw data were analysed in R using BeadArray and the Limma package. Ingenuity Pathway Analysis was used to perform pathway analysis on geneset data (IPA, Ingenuity Systems Inc. at www.ingenuity.com).

### RNA Isolation and Reverse-Transcription PCR (RT-PCR)

Total RNA was purified from cells using the Qiagen RNeasy Mini Kit (Qiagen) as per manufacturer’s instructions. First strand cDNA was synthesised from 1 to 2 μg of total RNA using M-MLV reverse transcriptase (Promega). RT-PCR was carried out on the Chromo4 System (Bio-Rad) using primers listed in Table S1. PCR conditions were as follows: 3 min at 95 °C and 40 cycles of 10 s at 95 °C, 30 s at 60 °C and 30 s at 72 °C followed by 10-s 70–95° melt curves. All experiments included three no-template controls and were performed on three biological replicates with three technical replicates for each sample and normalised to GAPDH. Results were analysed using Opticon Monitor software (Bio-Rad), and relative gene expression levels were calculated using the Pfaffl method [29].

### Chromatin Immunoprecipitation (ChIP) Analysis

ChIP was performed as described previously [30] and detailed here briefly. Cells were crosslinked with 1 % formaldehyde in PBS, quenched with 125 mM glycine and washed 3× with cold PBS (containing protease inhibitors) before centrifugation and lysis in lysis buffer [5 mM PIPES pH 8.0, 85 mM KCl, 0.5 % NP-40] for 30 min on ice. Pelleted nuclei were resuspended in shearing buffer [50 mM Tris pH 8.1, 10 mM EDTA, 0.1 % sodium dodecyl sulphate (SDS), 0.5 % sodium deoxycholate] and sonicated in a Bioruptor (Diogenode) with sufficient cycles (30 s on, 30 s off) to obtain an average chromatin shear size of 200–500 bp. Ten micrograms of pre-cleared chromatin was immunoprecipitated in modified RIPA buffer [140 mM NaCl, 10 mM Tris pH 7.5, 1 mM EDTA, 0.5 mM EGTA, 1 % TX-100, 0.01 % SDS, 0.1 % sodium deoxycholate] with specific antibodies (2 μg), protease inhibitors and pre-blocked magnetic protein G beads (Active Motif) at 4 °C. Following washes [2× Wash Buffer 1–20 mM Tris pH 8.1, 50 mM NaCl, 2 mM EDTA, 1 % TX-100, 0.1 % SDS; 1× Wash Buffer 2–10 mM Tris pH 8.1, 150 mM NaCl, 1 mM EDTA, 1 % NP-40, 1 % sodium deoxycholate, 250 mM LiCl; 2× TE], the chromatin was eluted [0.1 M NaHCO_3_, 1 % SDS] and de-crosslinked for 4 h at 65 °C with RNase and 200 mM NaCl then treated with proteinase K for 2 h at 42 °C. ChIP DNA was purified using a QIAquick PCR purification kit (Qiagen, according to manufacturer’s instructions). ChIP-qPCR was performed using ChIP DNA with promoter-specific primers (see Table S1). Controls included non-specific IgG and H3 ChIPs and ChIP-qPCR with primers for non-specific regions of genomic DNA where enrichment is not expected. Enrichment was analysed using a standard curve to quantitate, and data were normalised to total H3 (unless specified otherwise). qPCRs were run using an iCycler and MyiQ software (Bio-Rad).

The antibodies used were as follows: anti-H3 (rabbit IgG, AbCam), anti-H3K4me3 (rabbit IgG, AbCam; rabbit serum, Active Motif) and anti-H3K27me3 (rabbit IgG, Upstate) with rabbit IgG as a non-specific negative control. For primer sequences, see Table S1.

## Results

### Generation of Phenotypically Distinct Astrocytes from NPCs

**Fig. 1:**
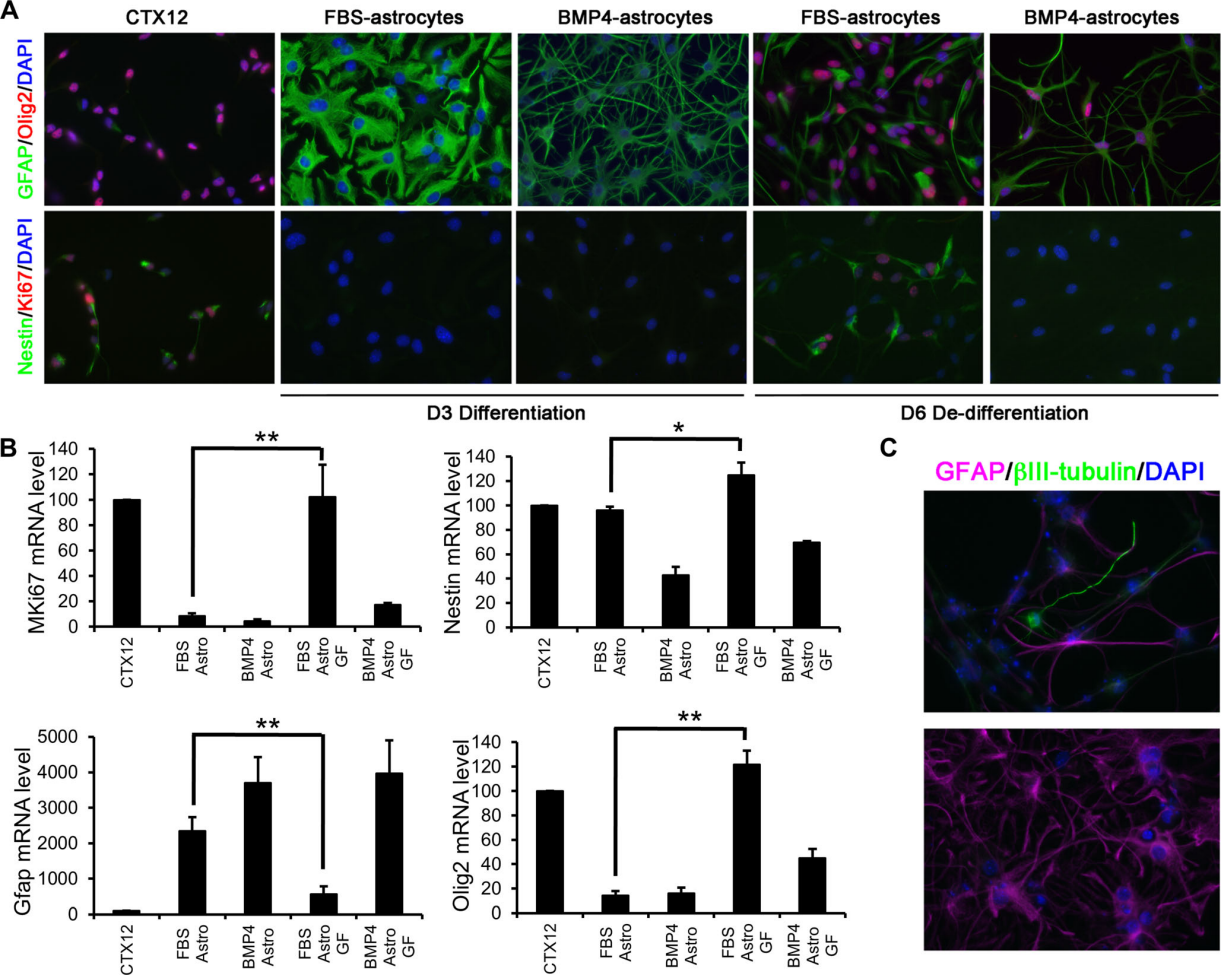
Molecular phenotype of CTX12 cells under proliferative conditions or in the presence of FBS or BMP4. **a** Immunocytochemistry showing GFAP (*green*) and Olig2 (*red*) expression (*top panels*) and Nestin (*green*) and Ki67 (*red*) expression (*bottom panels*). Nuclei are counterstained with DAPI (*blue*). CTX12s were differentiated for 3 days (*D3*) with either FBS or BMP4 followed by 3 days of dedifferentiation (*D6*). **b** Comparison of Mki67, Nes, GFAP and Olig2 gene expression in conditions shown in **a. c** Example of a βIII-tubulin-positive neuron (*green*) in FBS astrocyte cultures following dedifferentiation and tripotential differentiation (*top panel*). *Bottom panel* shows a representative image from BMP4 astrocytes under similar conditions (GFAP in *magenta* and DAPI in *blue*). Abbreviations: *Astro*, astrocyte; *GF*, growth factors (FGF2/EGF/4-OHT). *Error bars* in **b** show SEMs, *n* = 3

The mouse NPC line, CTX12, grown as a monolayer in the presence of EGF, FGF2 and 4-OHT (collectively ‘GFs’) expresses classic NPC markers including Nestin and Sox2 (Figs. 1a, b and S1A) and can differentiate into both neurons and astrocytes (Fig. S1B, C). CTX12s cultured without GFs in the presence of FBS or BMP4 for 3 days led to astrocyte differentiation. In both conditions, CTX12 cells ceased proliferation (loss of Ki67), down-regulated Nestin and Olig2 and up-regulated GFAP (Fig. 1a, b). However, each condition generated homogeneous astrocytes with distinct morphology: FBS astrocytes were flatter with few processes whilst BMP4 astrocytes were more ramified and stellate (Fig. 1a). To test whether either population retained plasticity in an NPC-permissive environment, FBS/BMP4 were removed and replaced by GFs for 3 days (‘dedifferentiation’ conditions). FBS astrocytes became morphologically NPC-like, re-entered cell cycle, down-regulated GFAP and up-regulated Nestin and Olig2 (Fig. 1a, b). In contrast, few BMP4 astrocytes became proliferative, and morphology and gene expression were relatively unchanged (Fig. 1a, b). We subjected dedifferentiated cells to a tripotential differentiation paradigm [31] to test their ability to generate neurons and glia. Both βIII-tubulin-positive neurons and GFAP-positive astrocytes were readily obtainable from FBS astrocytes, but almost none were generated from BMP4 astrocytes (Figs. 1c and S1D). Thus, only FBS astrocytes re-acquire an NPC state following dedifferentiation, suggesting that they differ in their neurogenic potential from the BMP4 astrocytes.

### Identification of a Common Set of Astrocyte-Regulated Genes and Pathways

To identify pathways or factors that regulate astrocyte differentiation and potential in our cellular model, we performed microarray analysis on CTX12s, FBS and BMP4 astrocytes (GEO accession number, with the authors). We identified ~8,000 probes with significantly changed expression upon differentiation from NPCs to FBS or BMP4 astrocytes (6,326 and 6,256 genes, respectively, false discovery rate (FDR) < 0.05, *Benjamini*-*Hochberg* [32], Tables 1 and Table S2, qPCR validation in Fig. S2). We first focussed on changes and enriched pathways common to both astrocyte populations following differentiation. Classical astrocyte markers were highly up-regulated including GFAP, Aqp4 and Slc39a12 [33]. Pathway analysis (IPA, Ingenuity Systems, Inc.) following FBS or BMP4 differentiation revealed a remarkable overlap in the most significant results, including cell cycle and proliferation-associated functions (Table S3), several of which are known to be enriched in astrocytes [33]. IPA also permits prediction of upstream regulators and their activation state, with several common to BMP4 and FBS astrocytes (relative to CTX12) including activation of TP53 and NFκB-related regulators and inhibition of MYC (Table S3).

**Table 1:** Gene expression comparison between CTX12 and FBS/BMP4 astrocytes

Gene symbol	FBS astrocytes		BMP4 astrocytes		Description
	Rank	Log2 fold change	Rank	Log2 fold change	
Up					
GFAP^a, b^	5	8.54	1	9.85	Glial fibrillary acidic protein
Aqp4^b^	6	8.53	2	8.64	Aquaporin 4
Clu	7	8.04	5	7.27	Clusterin
Socs3	33	5.23	34	4.92	Suppressor of cytokine signalling 3
Smad6	88	4.25	254	2.96	MAD homolog 6
Id2	151	3.75	112	3.75	Inhibitor of DNA binding 2
Pygb	191	3.39	99	3.89	Brain glycogen phosphorylase
Klf2	262	3.04	–	–	Krüppel-like factor 2
Nanog	835	1.88	731	1.9	Nanog homeobox
Vim	1,033	1.67	1,626	1.11	Vimentin
Stat1	1,229	1.50	1,070	1.54	Signal transducer and activator of transcription 1
Jak1	1,467	1.3	932	1.65	Janus kinase 1
Smad1	1,849	1.06	2,134	0.84	MAD homolog 1
Stat3	1,854	1.05	2,414	0.72	Signal transducer and activator of transcription 3
Sox2^a, c^	2,059	0.95	1,027	1.57	SRY-box-containing gene 2
Aldh1L1	2,174	0.88	1,126	1.49	Aldehyde dehydrogenase 1 family, member L1
S100β	–	–	464	2.33	S100 protein beta polypeptide
Down					
Ccnb1^c^	4	−8.41	4	−8.27	Cyclin B1
Cdc20	14	−7.2	11	−7.3	Cell division cycle 20
Jag1	176	−3.75	253	−3.17	Jagged 1
Olig2^a, b^	183	−3.70	162	−3.79	Oligodendrocyte transcription factor 2
Sox8	238	−3.39	462	−2.22	SRY-box-containing gene 8
Hes5	374	−2.74	611	−1.92	Hairy and enhancer of split 5
Ccnd2	530	−2.27	444	−2.29	Cyclin D2
Pdgfra	1,816	−0.91	860	−1.57	Platelet-derived growth factor receptor alpha

Selected significantly up- and down-regulated genes identified by Illumina BeadChip array from over 6,000 significantly changed genes (FDR<0.05). Rank denotes gene position in the up- or down-regulated gene lists ordered from highest to lowest fold change. Log2 change is the expression fold change in FBS- or BMP4-derived astrocytes relative to CTX12 cells

*ChIP* chromatin immunoprecipitation

^a^Genes validated by immunofluorescence

^b^Genes validated by qPCR

^c^Genes validated by ChIP

### Identification of a Set of Differentially Regulated Astrocyte Genes

**Fig. 2:**
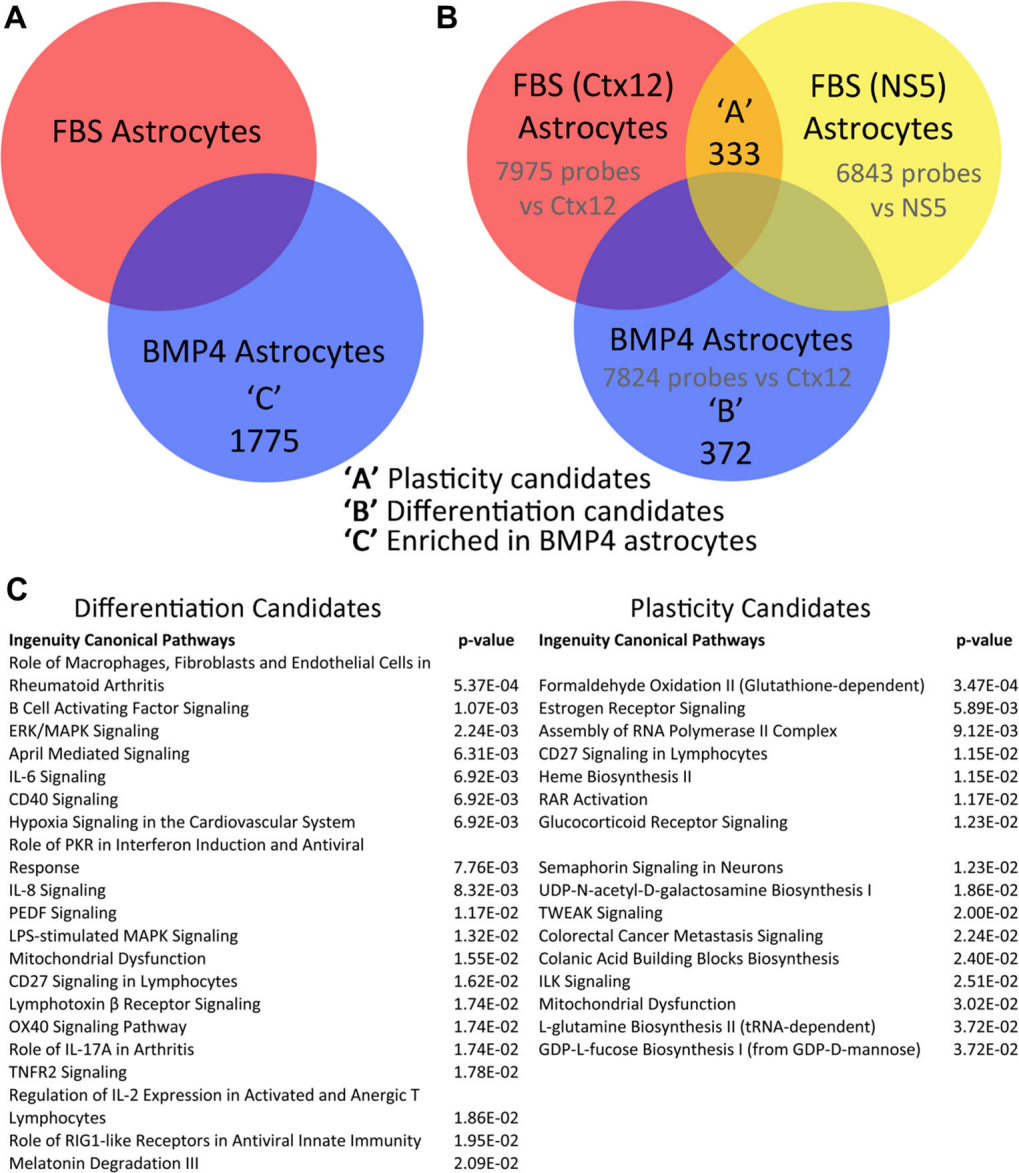
Candidate genes and signalling pathways involved in phenotype and potential of specific astrocyte populations. **a** Venn diagram showing number of genes expressed at a significantly different level between BMP4 and FBS astrocytes. **b** Venn diagram showing genes whose expressions were significantly changed in FBS- or BMP4-derived astrocytes compared to undifferentiated CTX12 or NS5 cells (FDR < 0.05). **c** Top canonical pathways enriched in the differentiation candidate gene set ‘*B*’ and ‘plasticity’ candidate gene set ‘*A*’ both shown in **b**

Next, we focussed on genes differentially expressed between BMP4 and FBS astrocytes and identified 1,775 probes (1,579 genes, Fig. 2a and Table S4, FDR < 0.05, *Benjamini*-*Hochberg*) including genes known to be enriched in specific astrocyte populations (Table S5) [33–35]. We first wanted to identify candidates for maintenance of NSC/NPC and neurogenic properties in astrocytes. We compared differential gene expression between BMP4 and FBS astrocytes with that of a second, well-characterised NSC line, NS5 [36], and NS5-derived FBS astrocytes (GEO accession number XXXX), which have a similar phenotype to CTX12-derived FBS astrocytes (Fig. S3). We identified 372 ‘differentiation’ candidate genes (changed upon differentiation in BMP4 astrocytes only) and 333 ‘plasticity’ candidate genes (changed in FBS but not BMP4 differentiation, Fig. 2b and Table S6, FDR < 0.05). The most significant differentiation candidate was Mmd2, recently shown to be important in astrogliogenesis in vivo downstream of NFIA and Sox9 [37]. ‘Differentiation’ and ‘plasticity’ lists were used to infer over-represented signalling pathways (FDR < 0.05) where we found 46 and 16 pathways, respectively (Fig. 2c and Table S6). ‘Differentiation’ pathways included several linked with inflammation and reactive astrocytes, including pro-inflammatory (interleukin (IL)-1/6/8 and TNF-related) and anti-inflammatory (peroxisome proliferator-activated receptor (PPAR)) signalling [38–42]. Upstream regulators included the NFκB complex, which can be activated by upstream TNF signalling. In addition, inhibition of transforming growth factor (TGF)-β1 was highly significant between BMP4 and FBS astrocytes. Interestingly, BMP4 astrocytes had lower expression of the reactive astrocyte marker, Serpina3n [40]. Despite few enriched pathways in the ‘plasticity’ list, these too included inflammation as well as NFκB- and TNF-linked pathways such as TWEAK and ILK signalling, the latter of which is activated in reactive astrocytes [40] (Table S6). Thus, pathway analysis results from in vitro astrocytes suggested that inflammatory pathways might regulate NPC potential in astrocytes.

### Activation of NFκB by TNF-α Leads to Dedifferentiation of a Subset of BMP4 Astrocytes

**Fig. 3:**
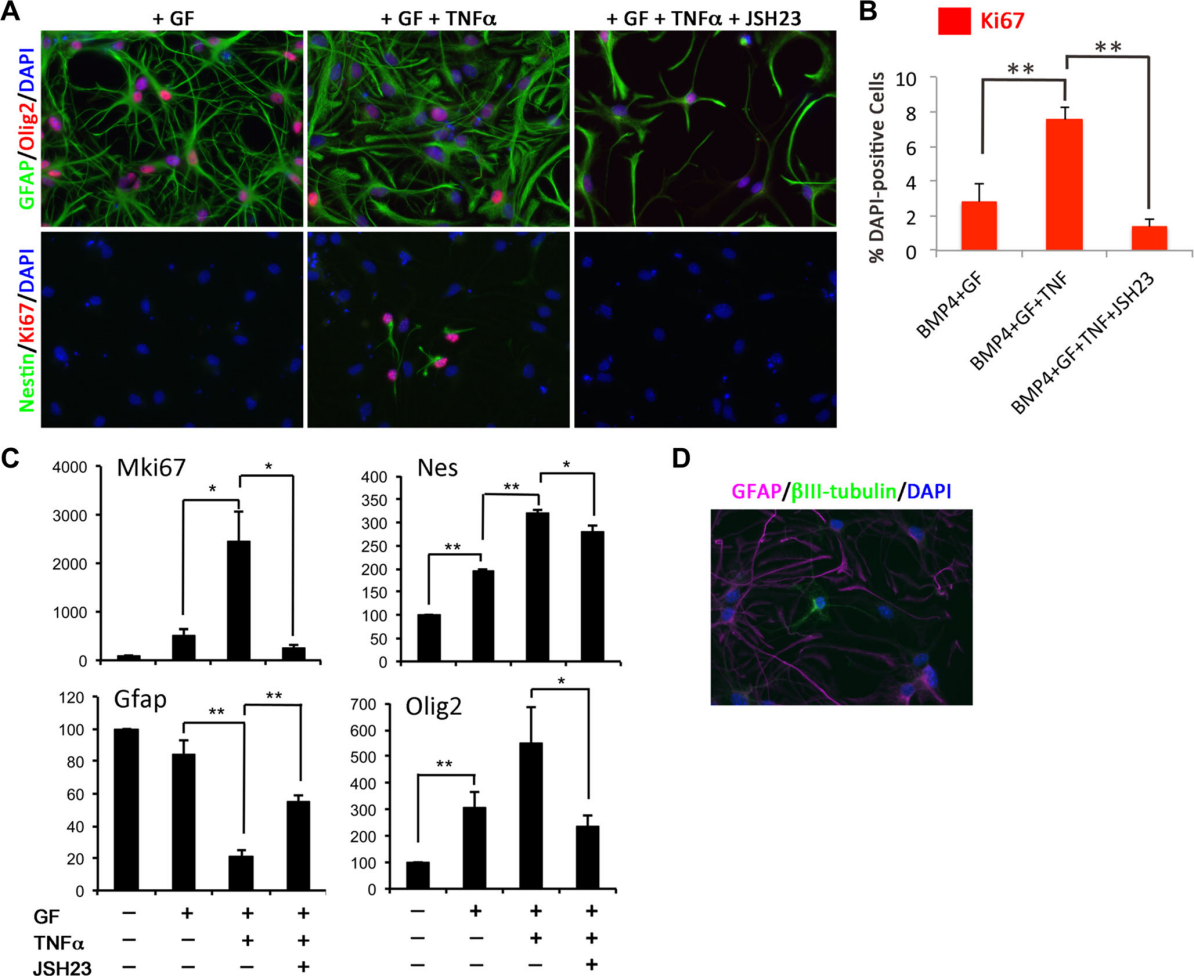
A subset of BMP4 astrocytes treated with TNF-α re-enter the cell cycle and re-acquire NPC characteristics. **a** Immunocytochemistry on BMP4 astrocytes cultured in dedifferentiation conditions for 3 days alone (GF), in the presence of TNF-α (+GF + TNFα) or with TNF-α and JSH23 (+GF + TNFα + JSH23). JSH23 was added 30 min before culturing astrocytes in dedifferentiation conditions in the presence of TNF-α. *Top row* shows GFAP (*green*) and Olig2 (*red*), and *bottom row* shows Nestin (*green*) and Ki67 (*red*). Nuclei were counterstained with DAPI (*blue*). **b** Percentage of Ki67-positive cells in cultures shown in **a. c** Gene expression levels of MKi67, Nestin (Nes), GFAP and Olig2 in BMP4 astrocytes in conditions shown in (**a**, **b**). Data are expressed as percentage of expression relative to D3 BMP4 astrocytes. **d** βIII-tubulin-positive neurons (*green*) and GFAP-positive astrocytes (magenta) with DAPI-labelled nuclei (*blue*) in BMP4 astrocyte cultures following dedifferentiation with TNF-α followed by tripotential differentiation. For **b** and **c**, *n* = 3. *Scale bars* in **a** and **d**: 20 μm. *P* values in **b** and **c**: *p < 0.05, **p < 0.01 (Student’s *t* test), *error bars* show SEMs

Under inflammatory conditions or following injury, mature parenchymal astrocytes can become reactive and re-acquire NPC-like properties [15]. Thus, we investigated the effect of activating the NFκB pathway, a common inflammatory signalling, on BMP4 astrocyte potential using TNF-α. The NFκB p65 subunit was largely cytoplasmic in BMP4 astrocytes but became nuclear following 2 h with TNF-α in proliferative conditions, confirming NFκB activation (Fig. S4A). BMP4 astrocytes in dedifferentiation conditions with TNF-α for 3 days showed a significant increase in mKi67, nestin and Olig2 and decrease in GFAP expression compared to growth factor (GF) alone and an increase in Ki67-positive cells (Fig. 3a–c), many of which became morphologically more NPC-like (Fig. 3a). A 24-h pulse of BrdU showed an increase in BrdU-positive cells with TNF-α (Fig. S4B, C). These effects were due to NFκB activation since they were reversed by the specific NFκB inhibitor, JSH-23 [43] (Fig. 3a, c). Cultures were subsequently tested for their neurogenic capacity, and a small number of ßIII-tubulin-positive neurons were observed with TNF-α (Fig. 3d). Therefore, NFκB activation through a TNF-α treatment induces a subset of BMP4 astrocytes to re-enter in the cell cycle and drives them to a more NPC-like state.

### TNF-α-Treated BMP4 Astrocytes Have a More NPC-Like Transcriptome

For a global view of TNF-α effects on BMP4 astrocytes, we performed microarray analysis to compare BMP4 astrocytes (BMP) with those in dedifferentiation conditions (GF) with or without TNF-α (TNF) (GEO accession number XXXX). We identified 3,712, 2,734 and 2,285 significantly changed genes comparing ‘TNF + GF v BMP4’, ‘TNF + GF v GF’ and ‘GF v BMP4’, respectively (FDR < 0.05, *Benjamini*-*Hochberg*, Table S7, qPCR validation in Fig. S5). Interestingly, the most significant up-regulated TNF gene, Lcn2, is a marker of reactive astrocytes in vivo [40]. TNF-α regulated genes (compared to BMP4 or GF) were enriched for cell cycle and growth and proliferation functions with pathways including control of cell cycle, p53, cancer-related pathways and TNF-related pathways (Table S8), consistent with a change in proliferative potential. Predicted upstream regulators included TNF and NFκB, activation of MYC, WNT and other pro-inflammatory molecules (including IFNG, IL1/17, Table S8). One of the few predicted regulators in the ‘GF v BMP’ dataset was sonic hedgehog (SHH) (activated), also in the TNF-α dataset, which may reflect the roles of pro-inflammatory molecules and SHH signalling in astrocyte acquisition of NSC properties [44, 45]. Interestingly, in dedifferentiation conditions (GF or TNF + GF), Ascl1, encoding the proneural protein Mash1, was up-regulated whilst members of Notch (Notch1, 4), Id (Id1-3), Stat (Stat2) and Hes (Hes1, 5) families were down-regulated, particularly in TNF + GF conditions. This is consistent with a neurogenic NPC-like capability since many are involved in the neurogenic–gliogenic switch, including several regulated by BMP signalling [46]. Taken together, these transcriptomic results highlight that the expression levels of several NPC-associated genes/pathways are more highly re-activated in the presence of TNF-α when compared to the corresponding BMP4-derived astrocytes under normal or dedifferentiation conditions.

### Mimicking CNS Damage Promotes NPC Properties in BMP4-Derived Astrocytes

**Fig. 4:**
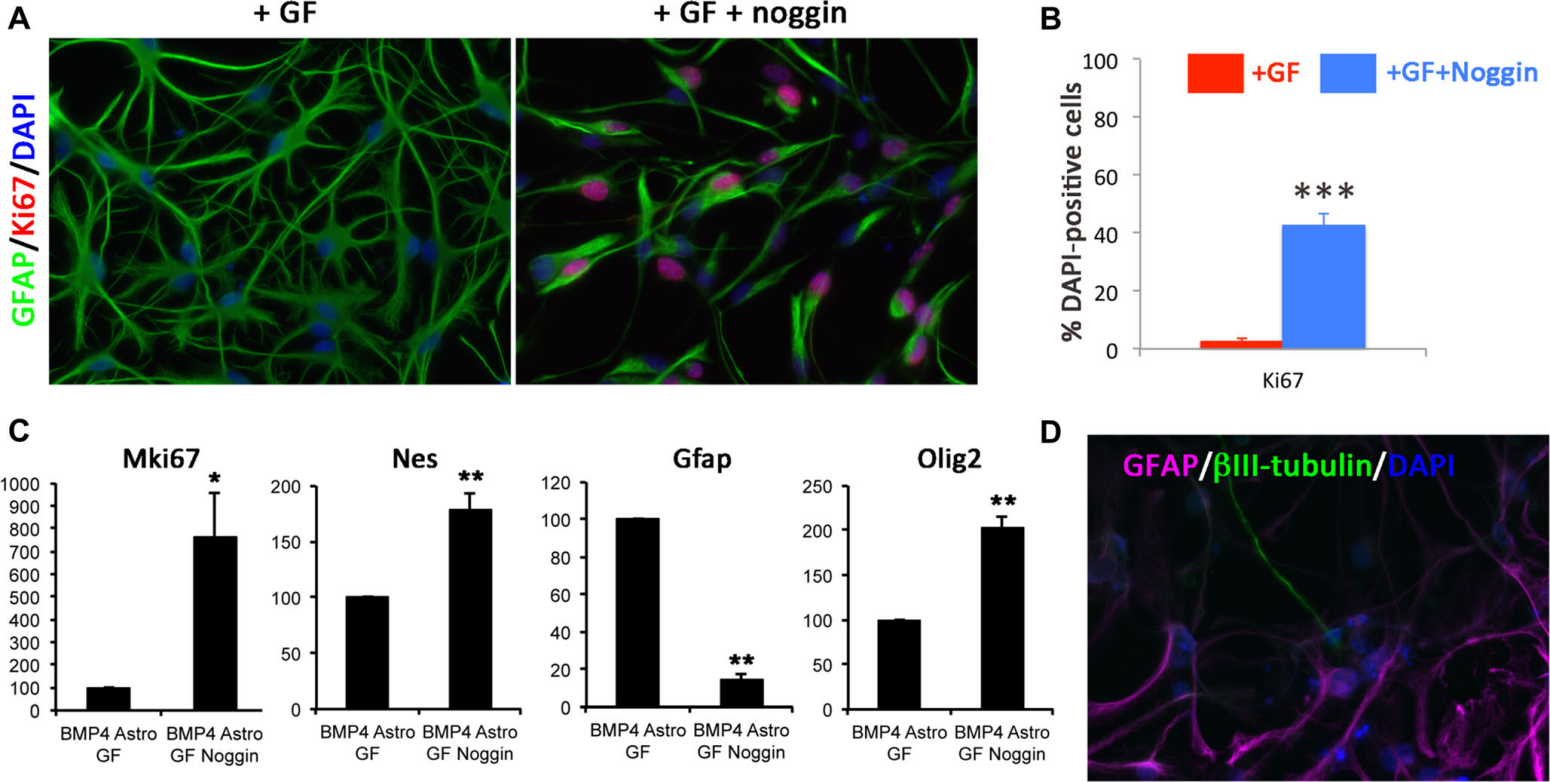
Inhibition of BMP signalling leads to re-acquisition of NPC characteristics in BMP4 astrocytes. **a** Immunocytochemistry comparing Ki67 (*red*) and GFAP (*green*) expression in BMP4 astrocytes in dedifferentiation conditions with and without noggin (+GF + noggin and +GF, respectively). Noggin was added simultaneously with GF. Nuclei are counterstained with DAPI (*blue*). **b** Percentage of Ki67-positive cells in the conditions shown in **a. c** Gene expression levels of Mki67, Nes, GFAP and Olig2 in the conditions shown in **a**. Expression levels are shown as percentage in +GF + noggin conditions relative to +GF conditions (100 %). **d** βIII-tubulin-positive neurons (*green*) and GFAP-positive astrocytes (*magenta*) with DAPI-labelled nuclei (*blue*) in BMP4 astrocyte cultures following dedifferentiation with noggin followed by tripotential differentiation. For **b** and **c**, *n* = 3. *Scale bars* in **a** and **d**, 20 μm. *P* values in **b** and **c**: *p < 0.05, **p < 0.01, ***p < 0.001 (Student’s *t* test), *error bars* show SEMs

In keeping with down-regulation of BMP-regulated genes, our analysis predicted inhibition of BMP itself under dedifferentiation conditions with and without TNF-α. This suggests that BMP4 astrocytes produce endogenous BMPs and that their inhibition is required for dedifferentiation. Interestingly, reactive astrocytes adjacent to penetrating CNS injuries in both spinal cord and brain up-regulate the BMP inhibitor, noggin [47], and BMP secreted from blood endothelial cells can induce reversible quiescence of NSC/NPCs in vitro, reversible with noggin [48]. We thus tested whether noggin could induce re-acquisition of NPC properties in BMP4 astrocytes. This led to a change from astrocyte to NPC-like morphology and a significant increase in proliferation (Fig. 4a, b), concomitant with down-regulation of GFAP and up-regulation of mKi67, Nestin and Olig2 (Fig. 4c) and down-regulation of genes enriched in parenchymal astrocytes, including Aqp4, Thrsp and Ngef (Table S5), to levels similar to dedifferentiated FBS-derived astrocytes (Fig. S6). Noggin-treated dedifferentiated astrocytes were also neurogenic, with a small number of ßIII-tubulin-positive neurons (Fig. 4d).

### Histone Modifications at Key Promoters Distinguish Between Astrocyte Types

Differentiation, including acquisition and maintenance of a more restricted fate, is known to be associated with epigenetic changes [20, 21, 24, 49]. The ability of FBS astrocytes to dedifferentiate compared to BMP4 astrocytes may reflect intrinsic epigenetic as well as transcriptional differences, including histone modifications in promoters associated with active (e.g. H3K9ac and H3K4me3) or repressed (H3K27me3) chromatin states [21, 50].

**Fig. 5:**
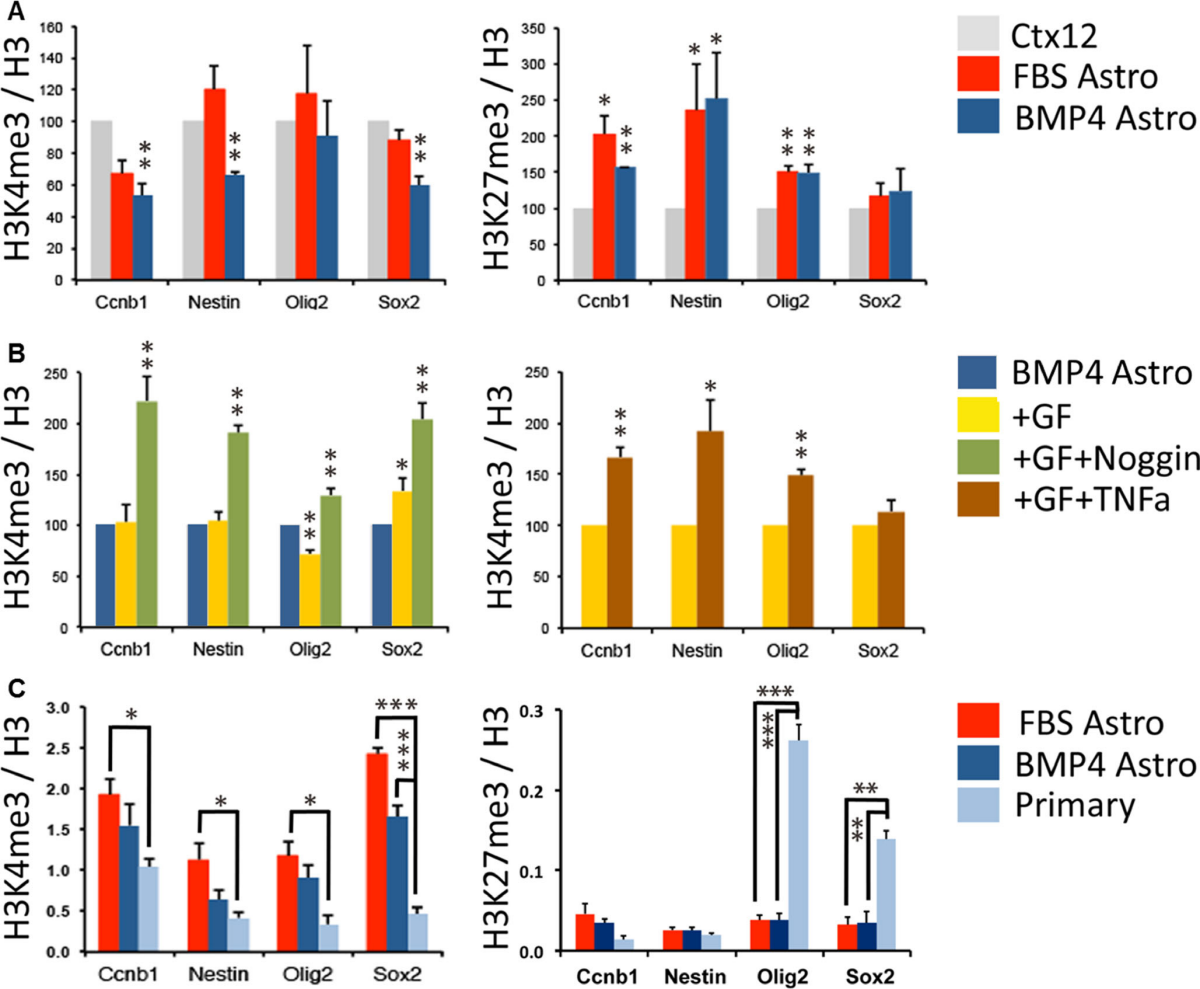
Epigenetic comparison of phenotypically distinct astrocytes. **a** H3K4me3 (*left*) and H3K27me3 (*right*) enrichment relative to total H3 at specific gene promoters in CTX12 cells (*grey*), FBS astrocytes (*red*) and BMP4 astrocytes (*blue*). Results are expressed as a percentage of levels in CTX12 (100 %). **b** *Left graph* shows H3K4me3 enrichment relative to total H3 at specific gene promoters in CTX12 cells (*grey*) and BMP4 astrocytes after 3 days of dedifferentiation alone (+GF, *yellow*) or with the addition of noggin (+GF + noggin, *green*). Results are expressed as a percentage of levels in CTX12 (100 %). The *right-hand graph* shows a comparison between BMP4 astrocytes in dedifferentiation conditions alone (+GF, *yellow*) and with TNF-α (+GF + TNFα). **c** Comparison of H3K4me3 (*left*) and H3K27me3 (*right*) enrichment in FBS astrocytes (*red*), BMP4 astrocytes (*dark blue*) and primary cortical astrocytes (*light blue*). For **a**–**c**, *n* = 3. *p < 0.05, **p < 0.01, ***p < 0.001 (Student’s *t* test), and *error bars* show SEMs

To look specifically at histone modifications associated with cellular potential, we used ChIP-qPCR to examine relative levels of H3K4me3 and H3K27me3 at selected gene promoters: *Ccnb1* (positive cell cycle regulator), *Nestin* (NPC-specific), *Olig2* (neural-specific bHLH factor) and *Sox2* (NSC/NPC marker). H3K4me3 was significantly decreased at *Ccnb1*, *Nestin* and *Sox2* in BMP4 astrocytes compared to CTX12s and FBS astrocytes, whilst H3K27me3 increased at *Ccnb1*, *Nestin* and *Olig2* in both astrocytes, corresponding with its down-regulation and cell cycle exit (Fig. 5a). Similar trends are shown at the Ascl1 locus, where H3K4me3 was significantly decreased in BMP4 astrocytes compared to CTX12s and FBS astrocytes along with H3K27me3 increase in both astrocytes (Fig. S7A, B). These data suggest that the epigenetic landscape reflects the differentiation state and may contribute to regulation of astrocyte plasticity. We therefore analysed H3K4me3 at the same promoters in TNF-α and noggin-treated BMP4 astrocytes in dedifferentiation conditions. With TNF-α, *Ccnb1*, *Nestin* and *Olig2* promoters showed increased levels of H3K4me3, whilst noggin-mediated dedifferentiation was accompanied by an increase at all four loci (Fig. 5b), suggesting a more permissive chromatin state. We next asked whether cultured primary astrocytes are associated with a less permissive chromatin state. We cultured P21 mouse cortical astrocytes and compared relative H3K4me3 and H3K27me3 levels with our in vitro astrocytes. P21 astrocytes had significantly lower H3K4me3 enrichment at all four loci compared to FBS astrocytes and *Sox2* in BMP4 astrocytes and higher H3K27me3 at *Sox2* and *Olig2* compared with both in vitro populations (Fig. 5c). Similar results were obtained at the Ascl1 locus, where H3K4me3 was significantly decreased, whilst H3K27me3 was increased in primary astrocytes compared to FBS- and BMP4-derived astrocytes (Fig. S7C, D). Although cultured parenchymal astrocytes may represent a more immature/reactive phenotype that non-cultured counterparts [33], our data are consistent with the restricted state of later postnatal astrocytes and suggest a more restricted state in BMP4 versus FBS astrocytes. Thus, astrocyte plasticity and neurogenic potential are reflected at the chromatin level.

### Temporal Changes in the Postnatal Astrocyte Transcriptome

**Fig. 6:**
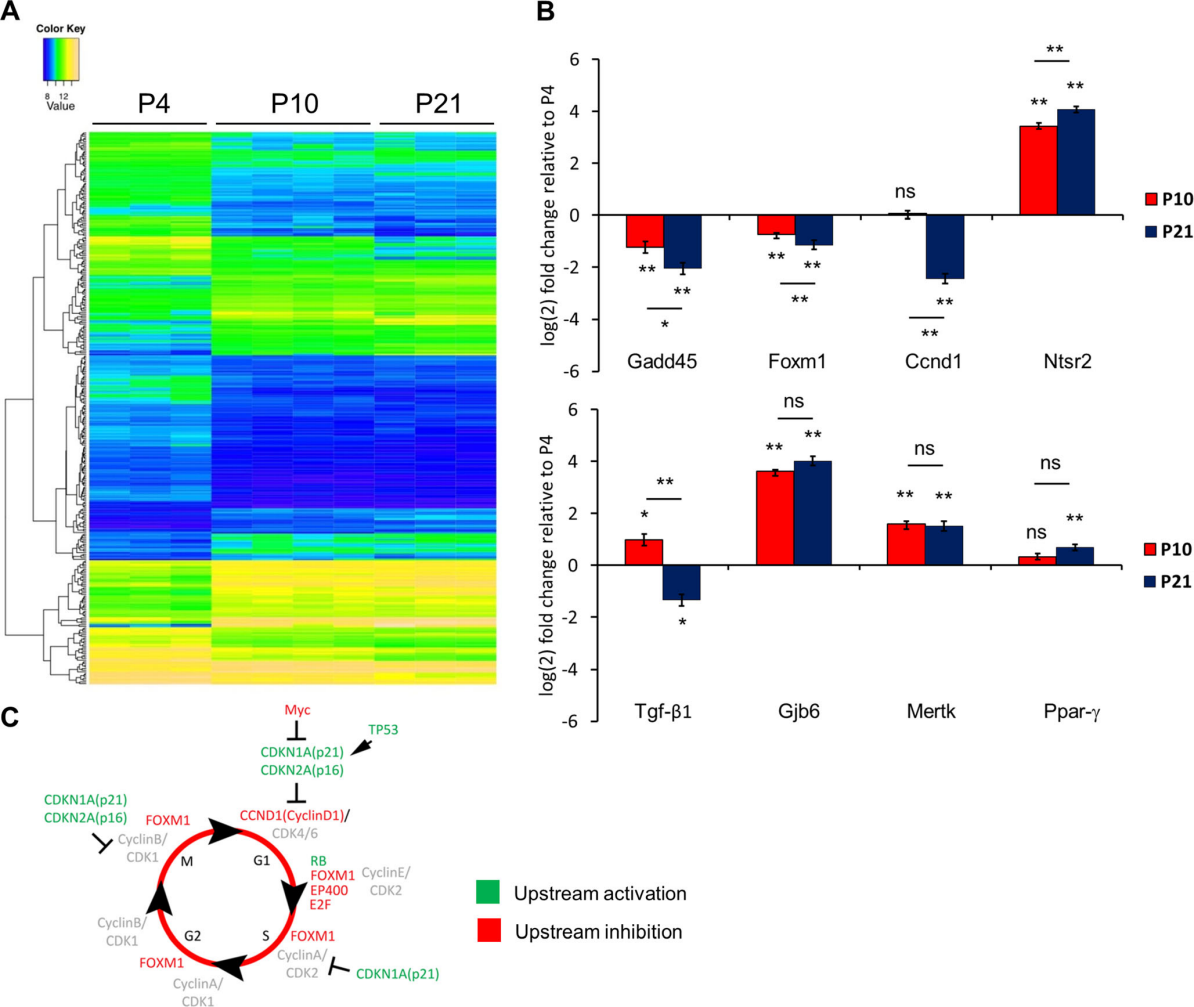
Developmental changes in gene expression in postnatal astrocytes. **a** Clustered heatmap showing relative gene expression levels in P4, P10 and P21 astrocytes from *Aldh1l1*-*EGFP* mice. Individual biological replicates are shown as individual columns for P4 (*n* = 3), P10 (*n* = 4) and P21 (*n* = 3). Relative expression levels are shown from low (*blue*) to high (*yellow*). **b** Graphs show Gadd45, Foxm1, Ccnd1, Ntsr2, TGF-β1, Gjb6, Mertk and PPARγ expression levels from FACSed *Aldh1l1*-*EGFP* astrocyte populations. Expression levels are shown in P10 (*red*) and P21 (*blue*) populations relative to P4 levels (*x*-axis). Data are the average of three biological replicates. *Error bars* show SEMs. *P* values: *p < 0.05, **p < 0.01, *ns* = not significant (Student’s *t* test). **c** Cell cycle diagram. The scheme shows activated (*green*) or inhibited (*red*) upstream regulators during in vivo astrocyte maturation as predicted by IPA

To further identify regulatory pathways responsible for astrocyte plasticity associated with specific differentiation states, we isolated ex vivo postnatal astrocytes by FACS from *Aldh1l1*-EGFP mice (Fig. S8A–C) [33], and we performed microarray analysis of astrocytes obtained from periods before, during and after which they lose neurosphere-forming potential (GEO accession number XXXX) [8]. We identified significantly changed genes (FDR < 0.05, *Benjamini*-*Hochberg*, qPCR validation in Fig. S9) from P4 to 10, P10 to 21 and P4 to 21 (Table S9) and determined functionally enriched pathways and regulators (Table S10). Most changes occurred from P4 to P10 (1,650 genes, Fig. 6a) with significantly enriched cell cycle and neurological disorder functions and cell cycle pathways and regulators (Fig. S10). Up-regulated genes also included Ntsr2, Gjb6 and Mertk (Fig. 6b), all highly enriched in mature in vivo astrocytes [33]. The top pathway identified was cell cycle regulation of replication. Interestingly, this was enriched in TNF + GF gene sets from BMP4 astrocytes, where many genes down-regulated from P4 to P10 showed up-regulation with TNF-α in vitro (Fig. S9). Signalling involving janus tyrosine kinase (JAK) family kinases and IL-6 revealed down-regulation of several genes by P10 that were significantly lower in BMP4 versus FBS astrocytes (Fig. S11). P10–P21 analysis (357 genes) again showed enrichment for cell cycle-associated pathways (including GADD45) and P4–P21 analysis (2,732 genes) identified additional pathways, some of which were identified in BMP4 astrocytes exposed to TNF-α (Tables S10 and S9). These data suggest common pathways in vitro and ex vivo that regulate astrocyte phenotype, potential and differentiation. We therefore interrogated upstream regulators to identify regulators of astrocyte differentiation and NSC potential.

P4–P10 upstream regulators included PPARG (and PPARGC1A), TP53 and CDKN2A activation and MYC, E2F1, EP400 and vascular endothelial growth factor (VEGF) inhibition (Table S10) with additional regulators from P10-P21 including FOXM1 (inhibition). Analysing P4–P21 also predicted CDKN1A activation and TGF-β1 and CCND1 inhibition. Broadly, these data support progressive cell cycle arrest (Fig. 6c) accompanied by changes consistent with inhibition of inflammatory signalling and identified other candidates with potential roles during early postnatal stages.

**Fig. 7:**
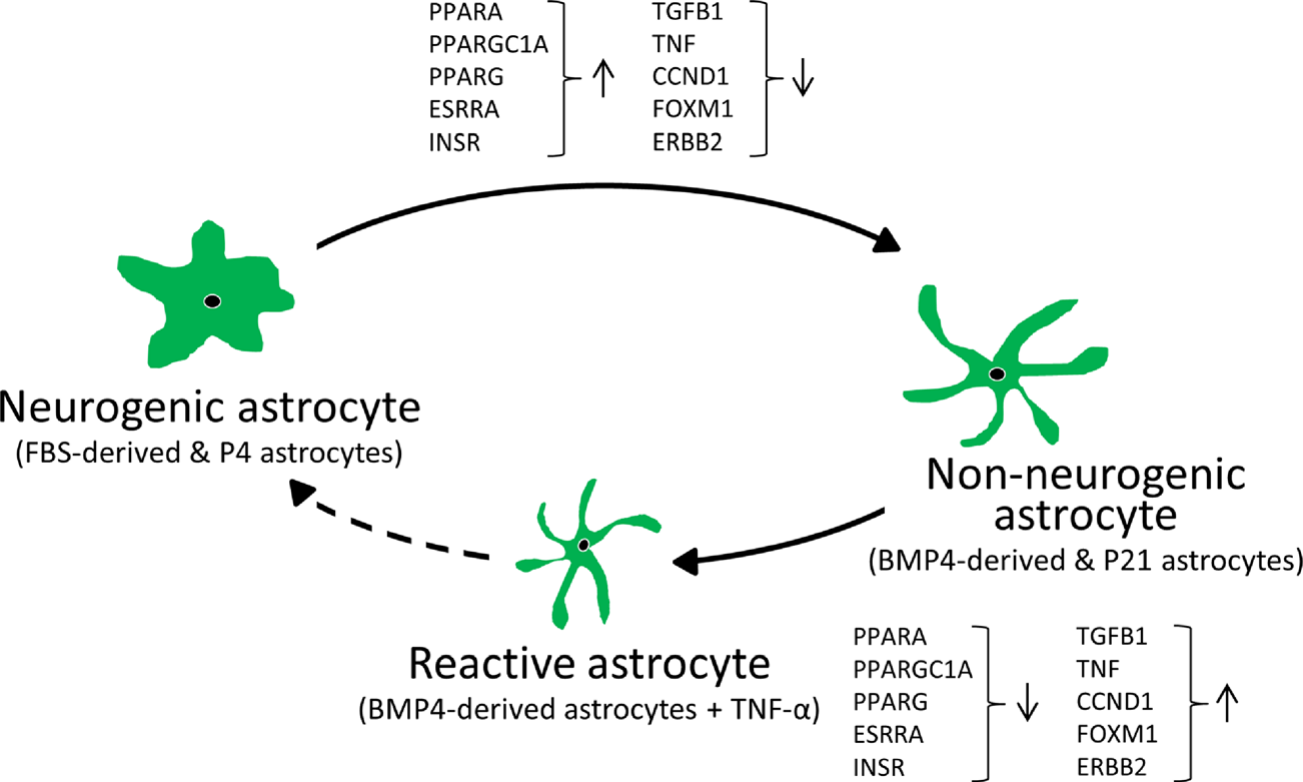
Candidate upstream regulators in the astrocyte neurogenic/non-neurogenic shift. The scheme illustrates that selected potential regulators that are responsible for the shift of neurogenic astrocytes (FBS-derived and P4 astrocytes) towards non-neurogenic astrocytes (BMP4-derived and P21 astrocytes) are also responsible for the dedifferentiation process under a pro-inflammatory environment (BMP4-derived astrocytes + TNF-α)

Intriguingly, many candidates followed the same pattern of inhibition/activation as BMP4 compared to FBS astrocytes or the opposing activation status following TNF-α exposure (Fig. 7 and Table S11). This further suggests that there are common, upstream regulators that control astrocyte differentiation as well as reactive phenotypes and dedifferentiation events in our in vitro and ex vivo models.

## Discussion

Adult neurogenesis occurs in two neurogenic niches from aNSCs with astrocyte-like characteristics [5, 51, 52]. Identification of glial progenitors outside these niches in healthy brain [53, 54] and reactive astrocytes with progenitor-like characteristics following injury [15] led to the notion that these cells may represent a source of progenitors for repair. In fact, recent studies have shown that stroke or excitotoxicity in mouse elicit a latent neurogenic program in striatal astrocytes [55, 56]. However, our current understanding of the key genes, pathways and mechanisms responsible for regulating astrocyte potential remains limited. Here, we describe two in vitro NSC-derived astrocytes populations that differ in their ability to dedifferentiate to an NPC-like state. We identified pathways associated with response to CNS injury and inflammation that regulate this differential potential and correlate with findings from ex vivo postnatal astrocytes. We also provide evidence for other putative regulators of parenchymal astrocyte development at postnatal stages when they acquire a terminally differentiated status and which may be involved in re-acquisition of NSC properties following injury. Finally, our data add to existing datasets from astrocyte populations that represent an invaluable resource to identify regulators of phenotype and potential towards developing therapeutic strategies for endogenous CNS repair.

### Inflammatory Pathways Regulate Astrocyte Neurogenic Potential and Differentiation

Continued expression of neurogenic fate determinants by cortical astrocytes at early postnatal stages ensures a gradual change from neurogenic to gliogenic RGCs and astrocytes [57, 58]. Interestingly, these findings are supported by previous studies showing that early postnatal astrocytes can dedifferentiate and give rise to neurogenic neurospheres, whilst this capacity declines during the second postnatal week [8]. Conceptually, functional specialisation is likely to require cell cycle exit and acquisition of a postmitotic status rendering astrocytes refractory to mitogens [59]. However, under inflammatory conditions or following injury, mature parenchymal astrocytes can become reactive and re-acquire immature or NPC-like properties [15, 60]. Nevertheless, signalling pathways that modulate astrogliosis with respect to time after injury and the type of damage are complex and not fully known [16, 61].

Here, we have used BMP4- and FBS-derived astrocytes to identify regulators that control the balance between astrocyte quiescence and maintenance of latent neurogenic potential. We identified a likely role for pro-inflammatory signalling, showing that NFκB activation by TNF-α can facilitate the re-acquisition of NPC properties. Indeed, our results showing that TNF-α treatment of BMP4-derived astrocytes modulates several Notch members (such as a decrease in Notch1 expression levels), are in agreement with the recent discovery that attenuated Notch1 signalling is necessary for neurogenesis by striatal astrocytes [55]. Several other signalling molecules including SHH and VEGF were also candidate regulators, which is interesting in light of recent evidence for the likely importance of signals from the vasculature for reactive astrocyte proliferation [62] and that SHH is necessary and sufficient to induce NSC-like properties in astrocytes [45]. It is also known that during brain injury, pro-inflammatory molecules, such as IL-1 and TNF-α, are produced [63] and activate SHH signalling and subsequent reactive gliosis in astrocytes [44]. It is intriguing to speculate that our in vitro model may recapitulate the action of a pro-inflammatory niche environment to activate SHH and VEGF in BMP4 astrocytes, leading to re-acquisition of NPC properties in a sub-population of cells. This is supported by our ex vivo parenchymal astrocyte expression data before and after the ‘neurogenic’ period with candidate upstream regulators including VEGF (inhibition). SHH signalling was previously reported as a significantly enriched pathway in postnatal astrocytes [33]. Our ex vivo data are also consistent with the hypothesis that SHH, VEGF and inflammatory-associated signalling may be involved in control of NSC properties in parenchymal, non-niche astrocytes during normal development and reactive gliosis.

Other pro- and anti-inflammatory pathway candidates identified included TGF-β1 and PPAR signalling, respectively. Based on our expression analyses, a more restricted (non-neurogenic) astrocyte phenotype correlates with inhibition of TGF-β1 and activation of PPAR (particularly PPARγ), and both of these pathways are amongst those highly enriched in postnatal astrocytes [33]. PPAR proteins (α, δ and γ) are nuclear hormone receptors with roles including regulation of inflammation in CNS disorders and following injury [39]. PPARα agonists can inhibit glial activation by lipopolysaccharides (LPS) by inhibiting astrocyte (and microglial) induction of TNF-α, IL-1β and IL-6, and PPARγ can inhibit NFκB and JAK/STAT signalling [38, 39]. PPARγ agonists can also affect the proliferation and differentiation of NSCs [64] and thus appear to be bona fide candidates for further investigation. TGF-β1 is a known mediator of inflammation whose expression is increased in the CNS in association with many disorders [42]. Exposure of postnatal parenchymal astrocytes to TGF-β1 in vitro causes widespread gene expression changes including up-regulation of the reactive astrocyte marker Lcn2, enrichment of the PPARα/RXRα activation pathway and association with immune or inflammation signalling (including NFκB and TNF) when used in combination with LPS and IFNγ [42]. Together, these data lend weight to the possibility of a cross-regulatory role between pro- and anti-inflammatory regulators that may be common to normal astrocyte differentiation (accompanied by loss of NSC properties) and re-acquisition of these properties following injury. Moreover, we have shown the validity of our in vitro system for identification of physiologically relevant candidate pathways.

### Changes in Cell Cycle Regulators Occur in the Postnatal ‘Neurogenic Window’

We identified few pathways or regulators linked with cell cycle as differentially represented between our two in vitro astrocytes; indeed, many were shared by both upon differentiation. For example, both showed inhibition of FOXM1 and activation of FOXO3—forkhead transcription factors with reciprocally antagonist actions implicated in many cancers and in cardiomyocyte proliferation [65, 66]. Furthermore, FOXO3 is also involved in regulation of NSCs, preventing premature neuronal differentiation [67, 68]. FOXM1 was significantly down-regulated during astrocyte maturation (from P4 to P21). Despite the heterogeneity of in vivo astrocyte populations, our ex vivo array data revealed a significant role for cell cycle-associated genes and regulators during maturation. This was particularly evident during the P4–P10 transition, after which the neurogenic capacity of parenchymal astrocytes is lost [8]. This included down-regulation of genes that have been previously shown to be decreased at P17 (Mcm2/5/6, Tgif2 and Uhrf1 [33]). Our observation that Stat3 and Jak2 are down-regulated between P4 and P10 (and are lower in BMP4 versus FBS astrocytes) implicate JAK kinases/IL-6 signalling in regulation of astrocyte plasticity. This is supported by their up-regulation during induction of astrogliosis [69].

### Endogenous BMP Signalling Is Able to Maintain In Vitro Astrocyte Quiescence

Following CNS injury, reactive astrocytes can up-regulate noggin expression [47]. Exogenous noggin in our in vitro model increased proliferation and acquisition of a more NPC-like phenotype in BMP4 astrocytes, showing that endogenous BMP signalling may regulate their proliferative and neurogenic properties. Indeed, increased levels of secreted BMPs upon loss of p21 in adult NSCs lead to their premature differentiation into mature astrocytes [70]. However, expression of Bmp and noggin was not significantly different between BMP4 and FBS astrocytes (except Bmp1 is lower in BMP4 astrocytes). Serum is often used as a proxy for BMPs; however, different levels of BMPs can affect different cellular responses. For example, low BMP2 levels increase proliferation of embryonic NPCs, whilst higher levels induce differentiation [71]. Interestingly, this is due to the differential activation status of the BMP receptor BMPR1B (relative to BMPR1A), which at higher levels leads to cell cycle arrest (and apoptosis or terminal differentiation). Indeed, BMPR1A and 1B have directly opposing roles (positive and negative, respectively) in regulating astrogliosis in vivo [72]. BMP4 astrocytes express twofold higher Bmpr1b and significantly lower Bmpr1a than FBS astrocytes, a process that is reversed following dedifferentiation with TNF-α. Therefore, BMP4 and FBS astrocytes may have different responses to BMP signalling, and future work is required to test whether this explains noggin effects on astrocyte potential.

### The Epigenetic Profile of Astrocytes Correlates with Their Potential

The chromatin landscape acts as a cellular memory that determines and permits long-lasting transcriptional programmes throughout development and differentiation [73]. Changes in H3K4me3 and H3K27me3 are coordinately controlled at genes activated or repressed, respectively, during differentiation [74, 75]. We have demonstrated that FBS astrocytes have more permissive chromatin at cell cycle- and NPC-related loci when compared to BMP4 astrocytes, correlating with their neurogenic potential. Moreover, TNF-α or noggin exposure induces the dedifferentiation of a subset of BMP4 astrocytes, and this is accompanied by epigenetic changes consistent with increased cellular potential. Of note, the importance of epigenetic regulation during neocortical development has been recently investigated, showing that the polycomb group complex (PcG) restricts neurogenic competence of NPCs and promotes the transition of NPC fate from neurogenic to astrogliogenic [76]. In this context, other studies in our group have shown that NSC-derived astrocytes retain an active epigenetic signature at promoters of neural lineage-specific genes, even though they are not expressed (unpublished data).

Our studies show that changes from neurogenic NPCs and astrocytes to non-neurogenic astrocytes are reflected at the transcriptional and epigenetic level. Further work will explore the relative contribution of identified inflammatory pathways and changes to the epigenome to astrocyte potential. Our data also add to existing datasets from astrocyte populations that represent an invaluable resource to identify regulators of phenotype and potential towards developing therapeutic strategies for endogenous CNS repair.

## Electronic Supplementary Materials

Below is the link to the electronic supplementary material.

ESM 1 (DOCX 38 kb)

Table S1 (XLSX 13 kb)

Table S2 (XLSX 7,604 kb)

Table S3 (XLS 1,683 kb)

Table S4 (XLSX 1,381 kb)

Table S5 (XLSX 43 kb)

Table S6 (XLSX 586 kb)

Table S7 (XLSX 1,525 kb)

Table S8 (XLS 2,217 kb)

Table S9 (XLSX 797 kb)

Table S10 (XLS 967 kb)

Table S11 (XLSX 12 kb)

Fig. S1 (GIF 148 kb)

High resolution (TIFF 1,358 kb)

Fig. S2 (GIF 34 kb)

High resolution (TIFF 346 kb)

Fig. S3 (GIF 206 kb)

High resolution (TIFF 1,856 kb)

Fig. S4 (GIF 152 kb)

High resolution (TIFF 1,560 kb)

Fig. S5 (GIF 48 kb)

High resolution (TIFF 505 kb)

Fig. S6 (GIF 44 kb)

High resolution (TIFF 454 kb)

Fig. S7 (GIF 53 kb)

High resolution (TIFF 189 kb)

Fig. S8 (GIF 287 kb)

High resolution (TIFF 4,037 kb)

Fig. S9 (GIF 37 kb)

High resolution (TIFF 136 kb)

Fig. S10 (GIF 104 kb)

High resolution (TIFF 1,790 kb)

Fig. S11 (GIF 64 kb)

High resolution (TIFF 948 kb)
