# Supplementary material for: The Neurogenic Potential of Astrocytes Is Regulated by Inflammatory Signals
Source: Mol Neurobiol. 2015 Jul 4;53(6):3724–39. doi: 10.1007/s12035-015-9296-x (PMC4937102; doi:10.1007/s12035-015-9296-x)
Supplement: Supplementary file 1 — (DOCX 38 kb) [file 12035_2015_9296_MOESM1_ESM.docx]

The neurogenic potential of astrocytes is regulated by inflammatory signals

Alessandro Michelucci^1,2*^, Angela Bithell^3*^^, Matthew J. Burney^1^, Caroline E. Johnston^1^, Kee-Yew Wong^4^, Siaw-Wei Teng^4^, Jyaysi Desai^1^, Nigel Gumbleton^3^, Gregory Anderson^1^, Lawrence W. Stanton^4^, Brenda P. Williams^1^, Noel J. Buckley^5^^

* These authors contributed equally to this work

^1^ King’s College London, Institute of Psychiatry, Centre for the Cellular Basis of Behaviour, The James Black Centre, 125 Coldharbour Lane, London, SE5 9NU, UK

^2^ Luxembourg Centre for Systems Biomedicine, University of Luxembourg, Campus Belval, 7, avenue des Hauts-Fourneaux, L-4362 Esch-Belval, G.D. Luxembourg (current address)

^3^ University of Reading, School of Pharmacy, Department of Pharmacology, The Hopkins Building, Whiteknights, Reading, RG6 6AP, UK

^4^ Genome Institute of Singapore, 60 Biopolis Street, #02-01, Genome Building, Singapore 138672

^5^ University of Oxford, Department of Psychiatry, Warneford Hospital, Warneford Lane, Oxford OX3 7JX, UK

**Supporting Information**

**Supplementary Tables**

**Table S1**. Primer sequences. Primer sequences for genotyping, qPCR gene expression analyses and ChIP-qPCR.

**Table S2**. Genes regulated during astrogliogenesis. Full list of significantly up-regulated and down-regulated genes from Illumina microarray data in FBS- and BMP4-astrocyte differentiation from CTX12 cells (FDR<0.05, *Benjamini-Hochberg*).

**Table S3**. IPA analysis including function, pathways and predicted regulators based on Illumina microarray data for BMP and FBS-astrocyte differentiation.

**Table S4**. Genes differentially expressed between FBS- and BMP4-astrocytes (FDR<0.05, *Benjamini-Hochberg*).

**Table S5**. Astrocyte enriched genes in multiple astrocyte populations.

**Table S6**. IPA analysis including function, pathways and regulators based on plasticity and differentiation candidate genes.

**Table S7**. Genes regulated by TNF-α. Full list of significantly up-regulated and down-regulated genes from Illumina microarray data in BMP4-astrocytes treated with TNF-α in dedifferentiating conditions (FDR<0.05, *Benjamini-Hochberg*).

**Table S8**. IPA analysis including function, pathways and regulators based on Illumina microarray data from BMP, BMP+GF and BMP+GF+TNF datasets.

**Table S9**. Genes regulated during astrogliogenesis *in vivo*. Full list of significantly up-regulated and down-regulated genes from Illumina microarray data from *Aldh1l1-EGFP* postnatal astrocytes (FDR<0.05, *Benjamini-Hochberg*).

**Table S10**. IPA analysis including function, pathways and regulators based on Illumina microarray data from P4, P10 and P21 astrocyte dataset comparisons.

**Table S11**. Candidate regulators of astrocyte potential from our study.

**Supplementary Figure legends**

**Figure S1. CTX12 differentiation into neurons and astrocytes.** (A) Immunofluorescence showing SOX2 expression in CTX12 cells (green). Nuclei are counterstained with DAPI (blue). (B) Immunofluorescence showing βIII-tubulin (green) and GFAP (magenta) expression in CTX12 cells following tripotential differentiation. Nuclei are counterstained with DAPI (blue). (C) Quantitation of cultures shown in (B). (D) Percentage of DAPI-positive cells positive for in FBS- and BMP4-astrocyte cultures following dedifferentiation (+GF) and subsequent tripotential differentiation (blue and red respectively). Data in (D) are the average of 3 biological replicates. P-values: *p<0.05 (Student’s *t*-test). Scale bar (A) & (B): 20µm.

**Figure S2. qPCR validation of CTX12, BMP4-astrocyte and FBS-astrocyte microarrays**. Graph shows validation of a range of genes across the dynamic range of the microarray data from CTX12 cells and both types of astrocytes (BMP4 and FBS). Expression levels are shown as log fold change (logFC) relative to CTX12 levels. Data are the average of 3 biological replicates (n=3). Error bars show SEMs. P-values: *p<0.05, **p<0.01, ***p<0.001, ns = not significant.

**Figure S3. Molecular phenotype of NS5 cells and NS5-derived astrocytes**. Immunofluoresence labeling from NS5 cells (left column) differentiated into astrocyte with FBS for 3 days (D3 astrocyte, middle column) then dedifferentiated for 3 days in EGF/FGF2 (D3 Dediff, right column). Top row shows expression of GFAP (green) and OLIG2 (red) and middle row shows expression of Nestin (green) and Ki67 (red). Nuclei are counterstained with DAPI (blue) in top 2 rows. Scale bar for immunofluorescence: 20μm.

**Figure S4. TNF-α activation of the NFκB pathway leads to dedifferentiation of a subset of BMP4-derived astrocytes**. (A) Immunofluorescence showing nuclear localisation of p65 in BMP4-astrocytes in dedifferentiating conditions (+GF) in the presence of TNF-α (white arrows). (B) Immunofluorescence showing BrdU labelling (green) with nuclei counterstained with DAPI (blue) in BMP4-astrocytes in dedifferentiating conditions alone (top) or with the addition of TNF-α (bottom). (C) Quantitation of (B) expressed as percentage BrdU-positive cells relative to total DAPI-positive cells. Scale bar (A): 20μm. Data in (C) represent the average from three biological replicates. **p<0.01 (using Student’s *t*-test) and error bars show SEMs.

**Figure S5. qPCR validation of BMP4-astrocyte dedifferentiation microarrays**. Graph shows qPCR validation for a number of genes across the dynamic range of the microarray data from BMP4-astrocytes alone (BMP), BMP4-astrocytes following 3 days in dedifferentiation conditions (BMP+GF) and in dedifferentiating conditions with TNF-α (BMP+GF+TNF). Expression levels are shown as log fold change (logFC) relative to CTX12 levels. Data are the average of 3 biological replicates (n=3). Error bars show SEMs. P-values: *p<0.05, **p<0.01, ***p<0.001, ns = not significant.

**Figure S6. qPCR analysis to assess the effect of noggin on gene expression during BMP4-astrocyte dedifferentiation**. Graph shows qPCR comparison for a number of genes in FBS-astrocytes following 3 days of dedifferentiation (FBS+GF) compared to BMP4-astrocytes following 3 days in dedifferentiation conditions with noggin (BMP+GF+Noggin). Expression levels are shown as log fold change (logFC) relative to CTX12 levels. Data are the average of 3 biological replicates (n=3). Error bars show SEMs. P-values: *p<0.05, ns = not significant.

**Figure S7. Epigenetic comparison of phenotypically distinct astrocytes at Ascl1 locus**. (A) H3K4me3 and (B) H3K27me3 enrichment relative to total H3 at Ascl1 gene promoter in CTX12 cells (grey), FBS-astrocytes (red) and BMP4-astrocytes (blue). Results are expressed as a percentage of levels in CTX12 (100%). (C-D) The graphs show a comparison of (C) H3K4me3 and (D) H3K27me3 enrichment in FBS-astrocytes (red), BMP4-astrocytes (dark blue) and primary cortical astrocytes (light blue). For (A-D) n=3. *p<0.05, **p<0.01 (Student’s *t*-test), and error bars show SEMs.

**Figure S8. Fluorescence activated cell sorting of pure astrocyte populations from *Aldh1l1-EGFP* mice**. (A) Immunofluorescence imaging of sections from Aldh1l1-EGFP transgenic mice (bottom panels) and wildtype littermates (top panels) at low (10x) and high (63x) magnification. Examples of EGFP-positive astrocytes in *Aldh1l1-EGFP* mice are shown by white arrows at higher magnification (63x). (B) Shows pre-FACS populations from Aldh1l1-EGFP mice sorted based on EGFP expression (top) and labeled by immunocytochemistry for EGFP (red) or GFAP (magenta) with DAPI-labelled nuclei (blue, bottom panels). (C) Shows pure, post-FACS astrocyte populations. Abbreviations: FACS, fluorescence activated cell sorting.

**Figure S9. qPCR validation of *Aldh1l1-EGFP* primary astrocyte microarrays**. Graph shows qPCR validation of a number of genes from FACSed *Aldh1l1-EGFP* astrocyte populations. Expression levels are shown in P10 (purple) and P21 (blue) populations relative to P4 levels (x-axis). Data are the average of 3 biological replicates. Error bars show SEMs. P-values: *p<0.05, **p<0.01, ns = not significant (Student’s *t*-test).

**Figure S10. Cell cycle control of replication pathway in primary and *in vitro*-derived astrocytes**. Contrasting gene expression changes with maturation state in primary astrocytes (P10 versus P4, left) and BMP4-astrocytes in pro-inflammatory (TNF-α) dedifferentiating conditions (TNF+GF versus GF alone, right). Figure shows a common, significantly over-represented pathway analysed using Ingenuity Pathways Analysis (IPA). Genes expressed at significantly lower or higher levels in P10 astrocytes (compared to P4) or BMP4-astrocytes with TNF+GF (compared to GF alone) are shown in green and red respectively.

**Figure S11. Jak family kinases in IL-6-type cytokine signaling pathway in primary and *in vitro*-derived astrocytes**. Similarities in gene expression changes with maturation state in primary astrocytes (P10 versus P4, left) and *in vitro* astrocytes (BMP4 versus FBS-astrocytes). Figure shows a common, significantly over-represented pathway analysed using Ingenuity Pathways Analysis (IPA). Genes expressed at significantly lower or higher levels in P10 astrocytes (compared to P4) or BMP4-astrocytes (compared to FBS) are shown in green and red respectively.
