# Supplementary figures and images for: The Neurogenic Potential of Astrocytes Is Regulated by Inflammatory Signals
Source: Mol Neurobiol. 2015 Jul 4;53(6):3724–39. doi: 10.1007/s12035-015-9296-x (PMC4937102; doi:10.1007/s12035-015-9296-x)

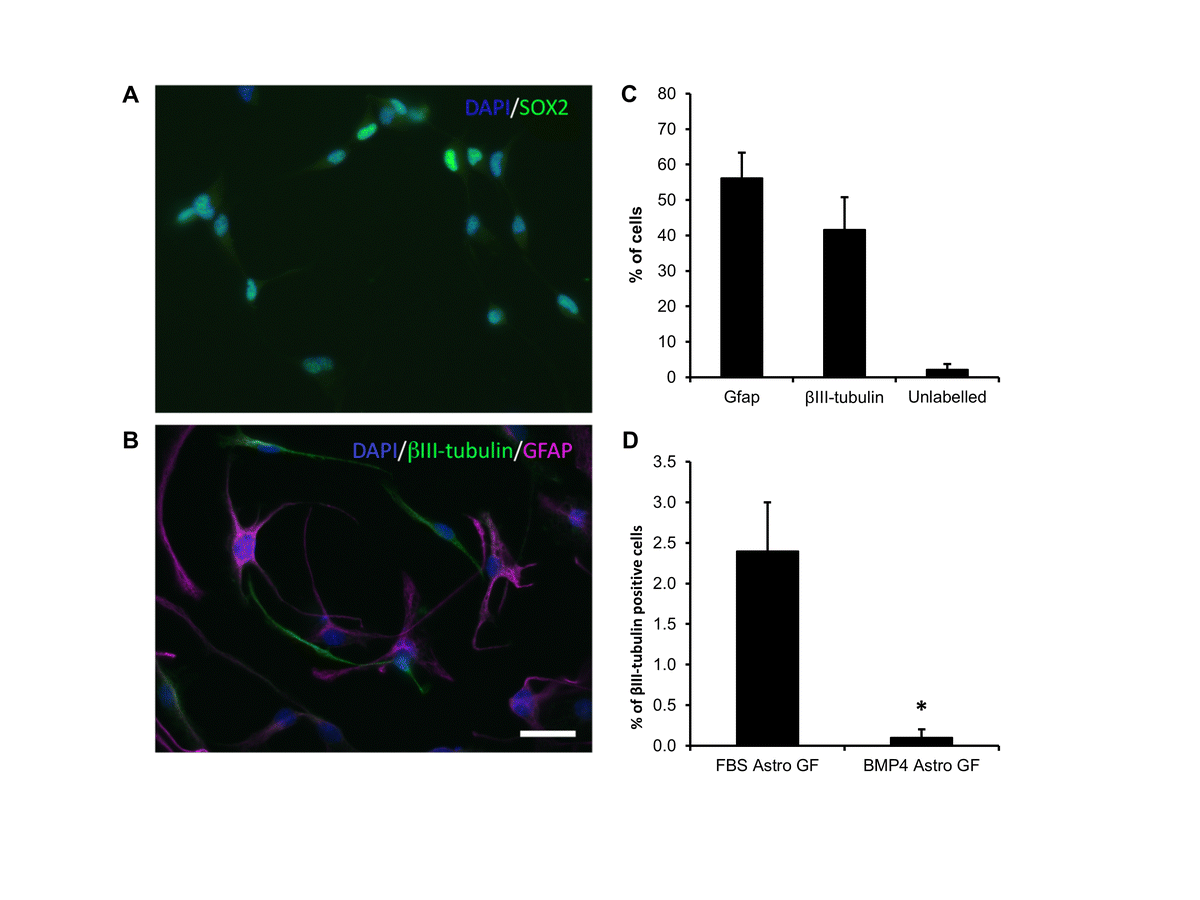

Supplement: Supplementary file 13 — (GIF 148 kb) [file 12035_2015_9296_Fig8_ESM.gif]

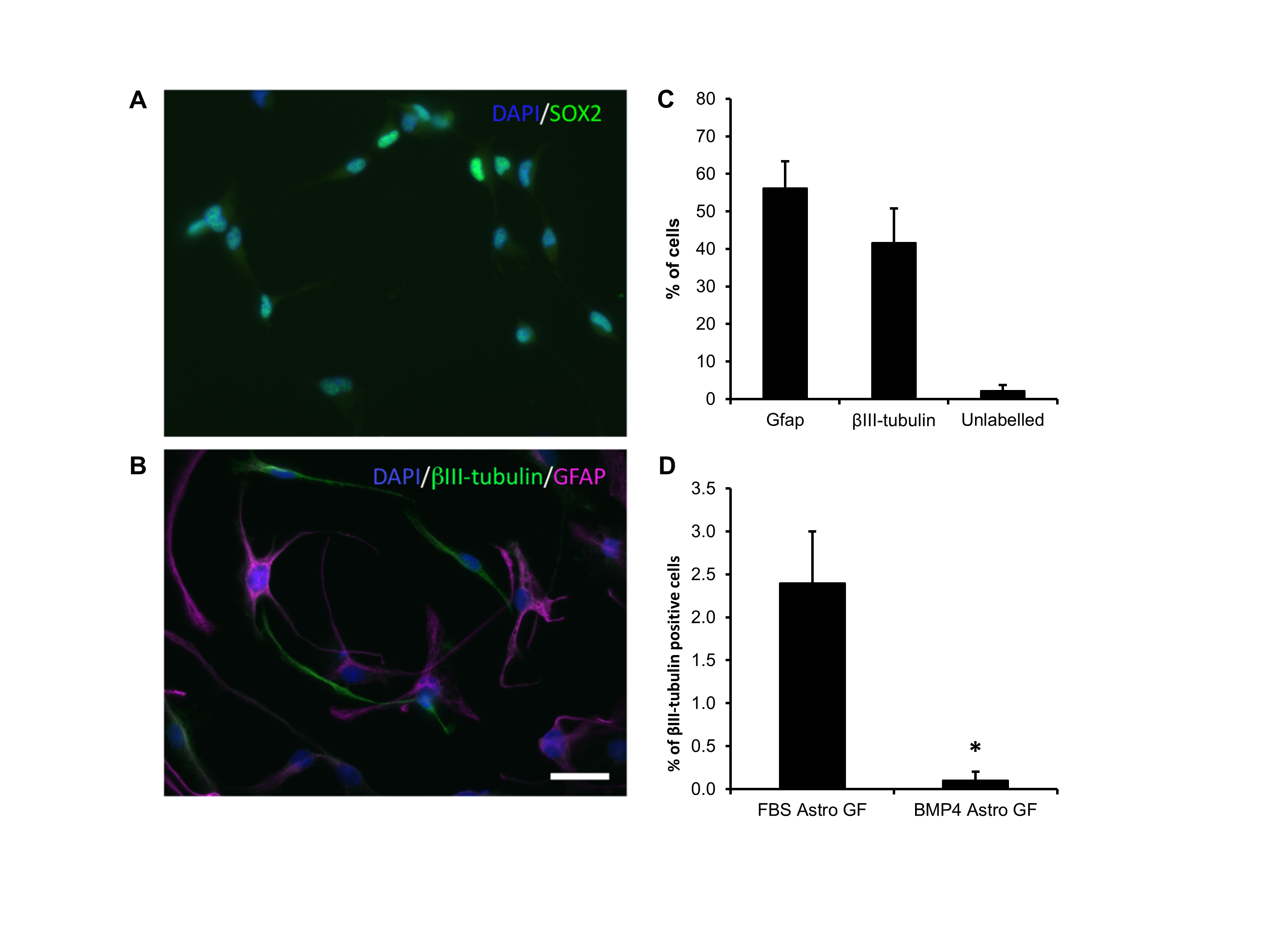

Supplement: Supplementary file 14 — High resolution (TIFF 1,358 kb) [file 12035_2015_9296_MOESM13_ESM.tif]

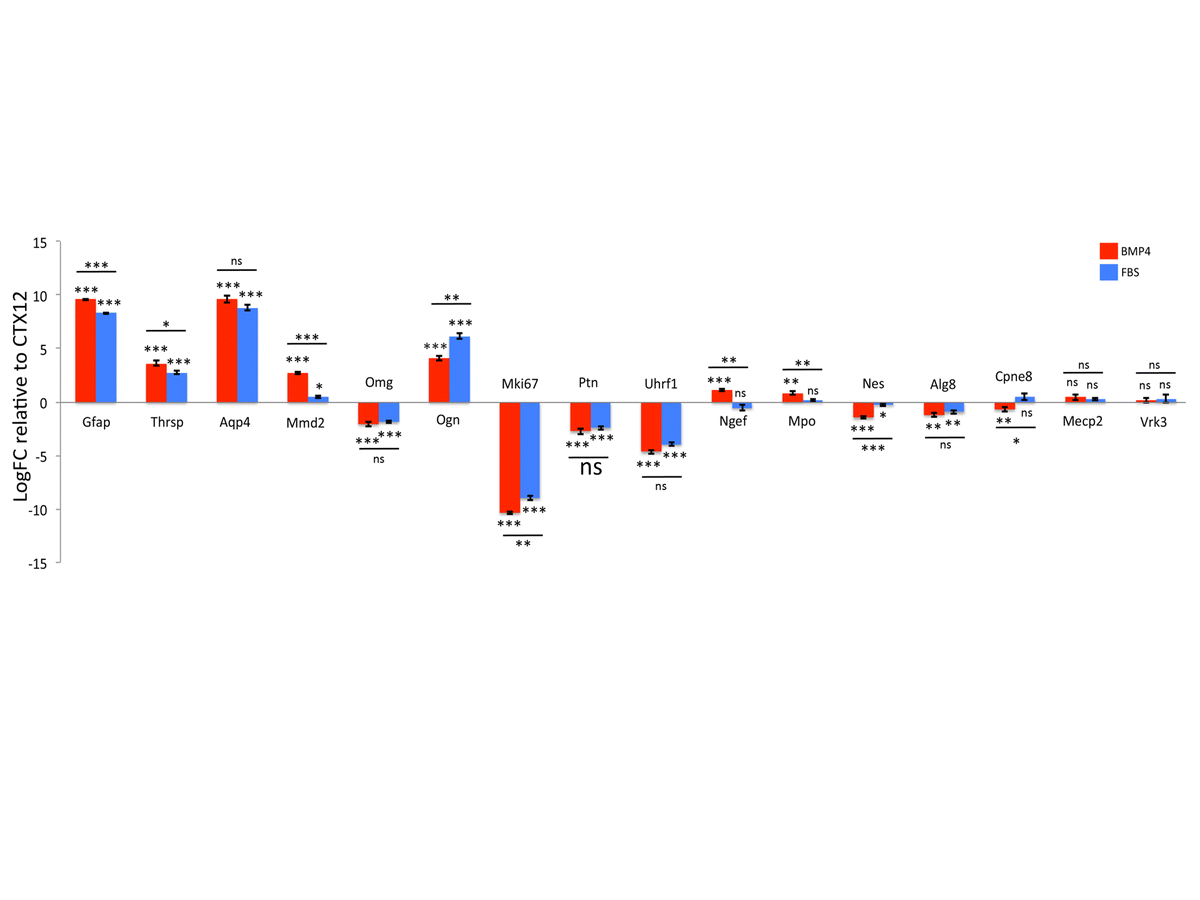

Supplement: Supplementary file 15 — (GIF 34 kb) [file 12035_2015_9296_Fig9_ESM.gif]

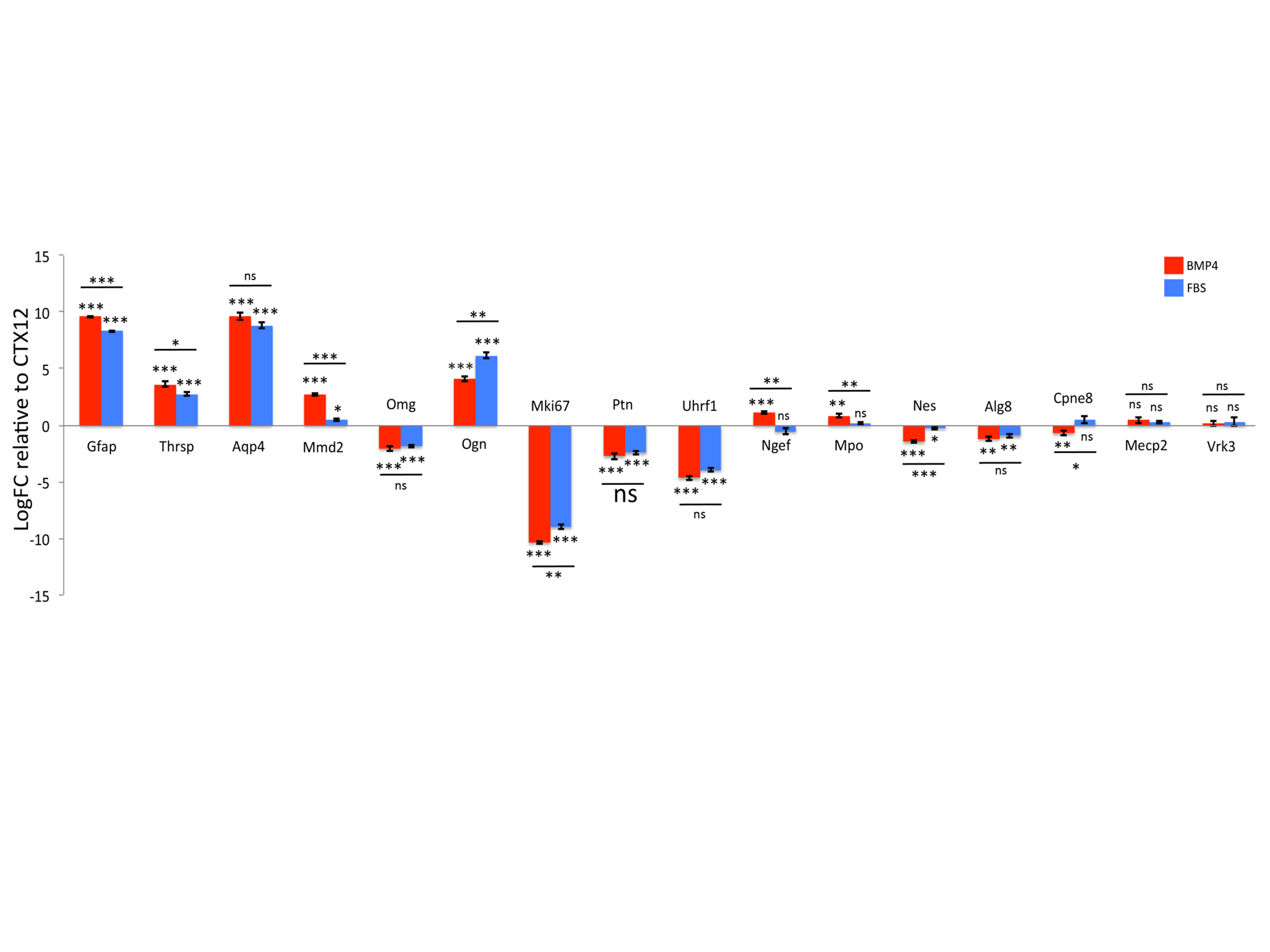

Supplement: Supplementary file 16 — High resolution (TIFF 346 kb) [file 12035_2015_9296_MOESM14_ESM.tif]

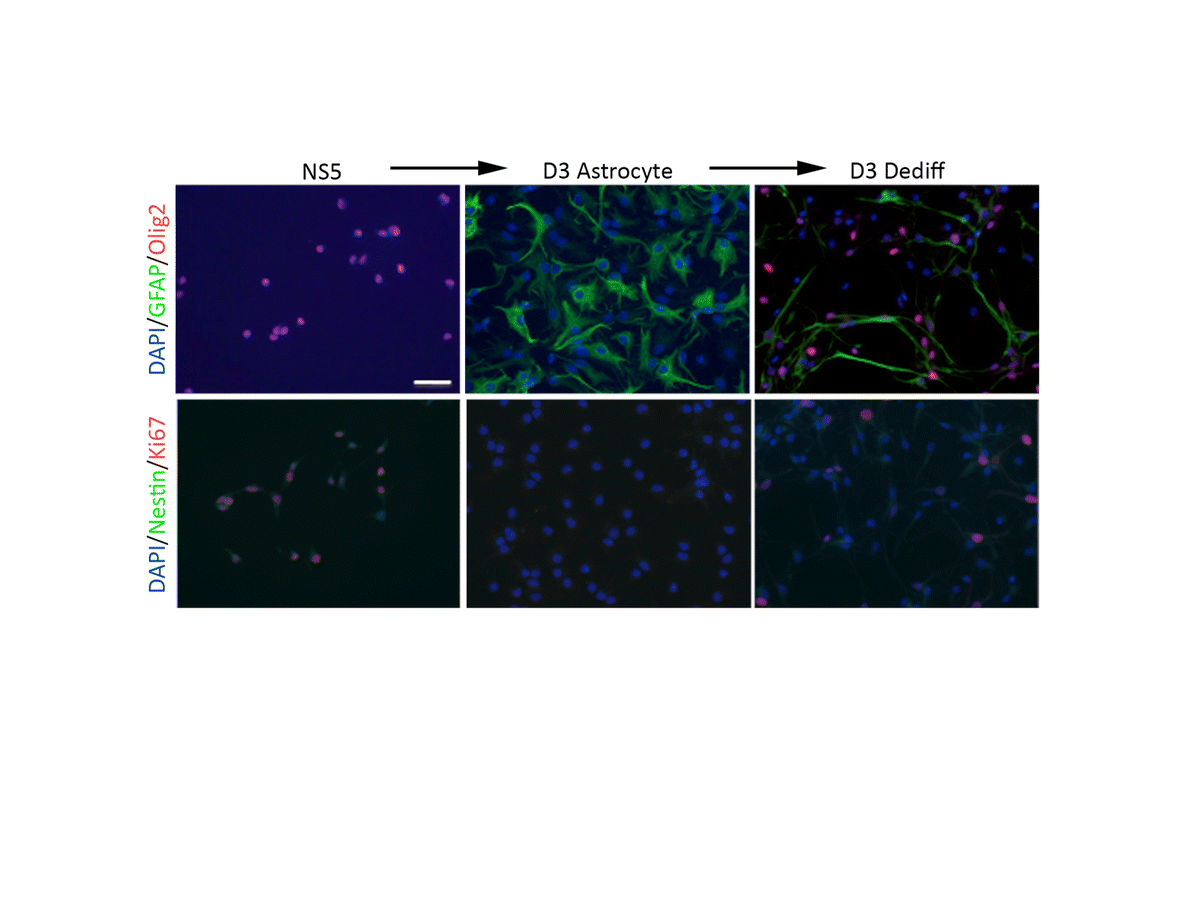

Supplement: Supplementary file 17 — (GIF 206 kb) [file 12035_2015_9296_Fig10_ESM.gif]

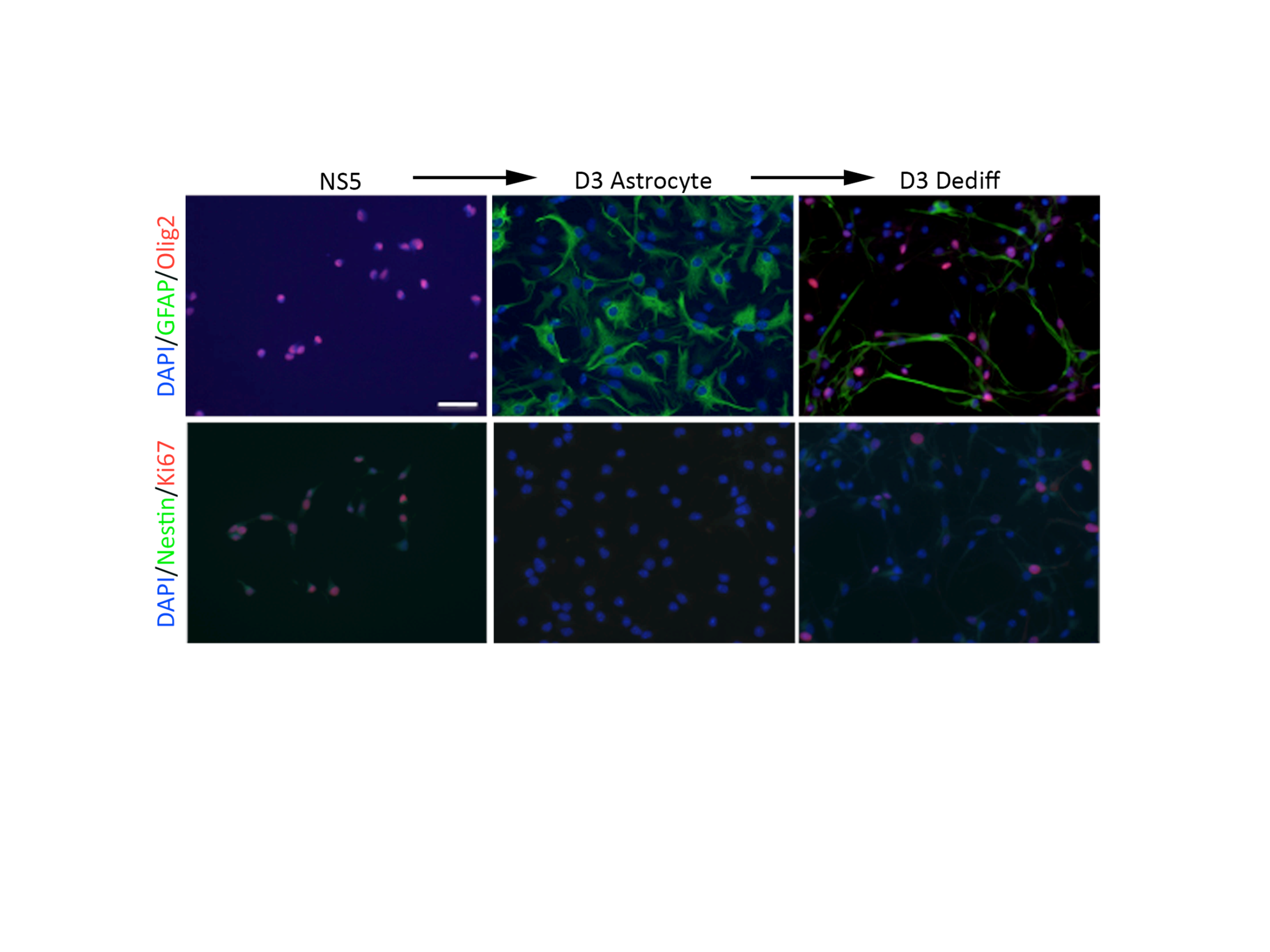

Supplement: Supplementary file 18 — High resolution (TIFF 1,856 kb) [file 12035_2015_9296_MOESM15_ESM.tif]

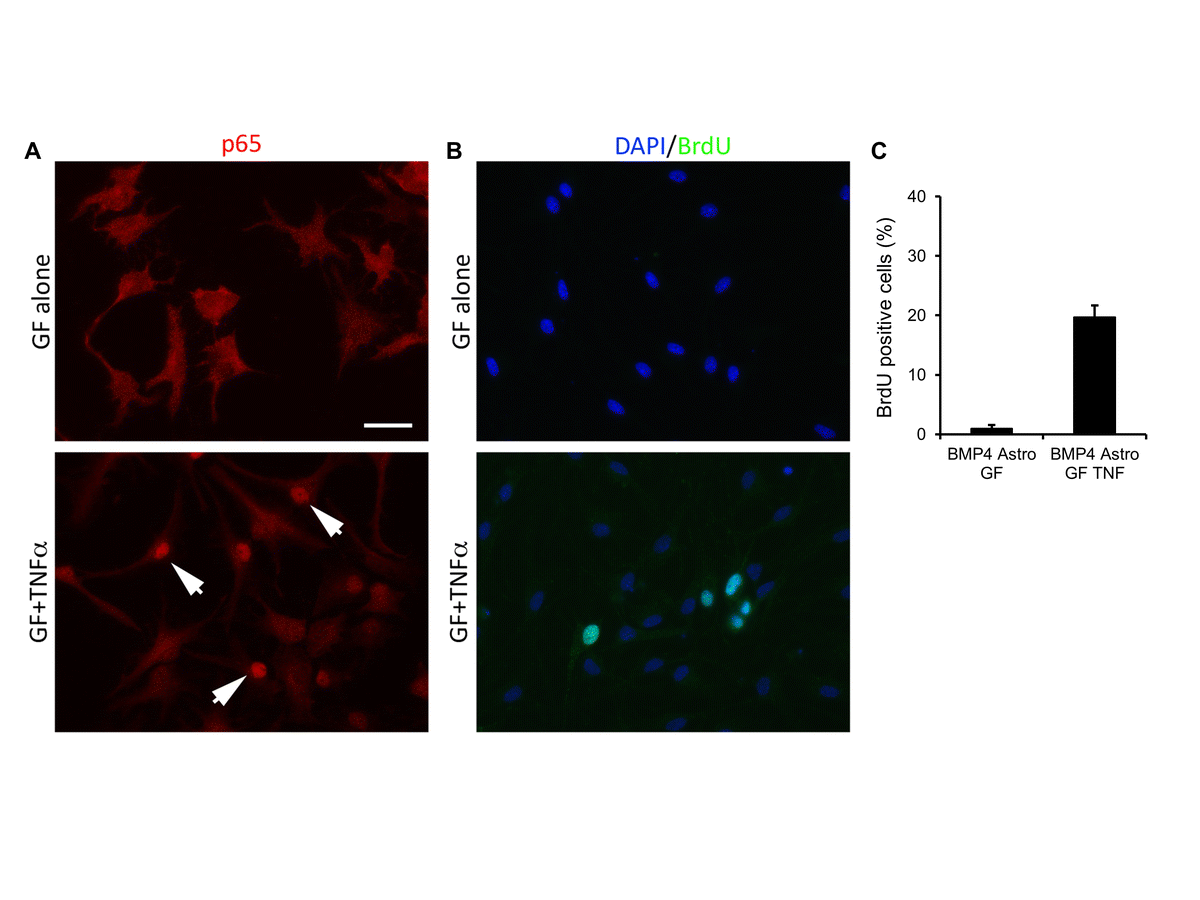

Supplement: Supplementary file 19 — (GIF 152 kb) [file 12035_2015_9296_Fig11_ESM.gif]

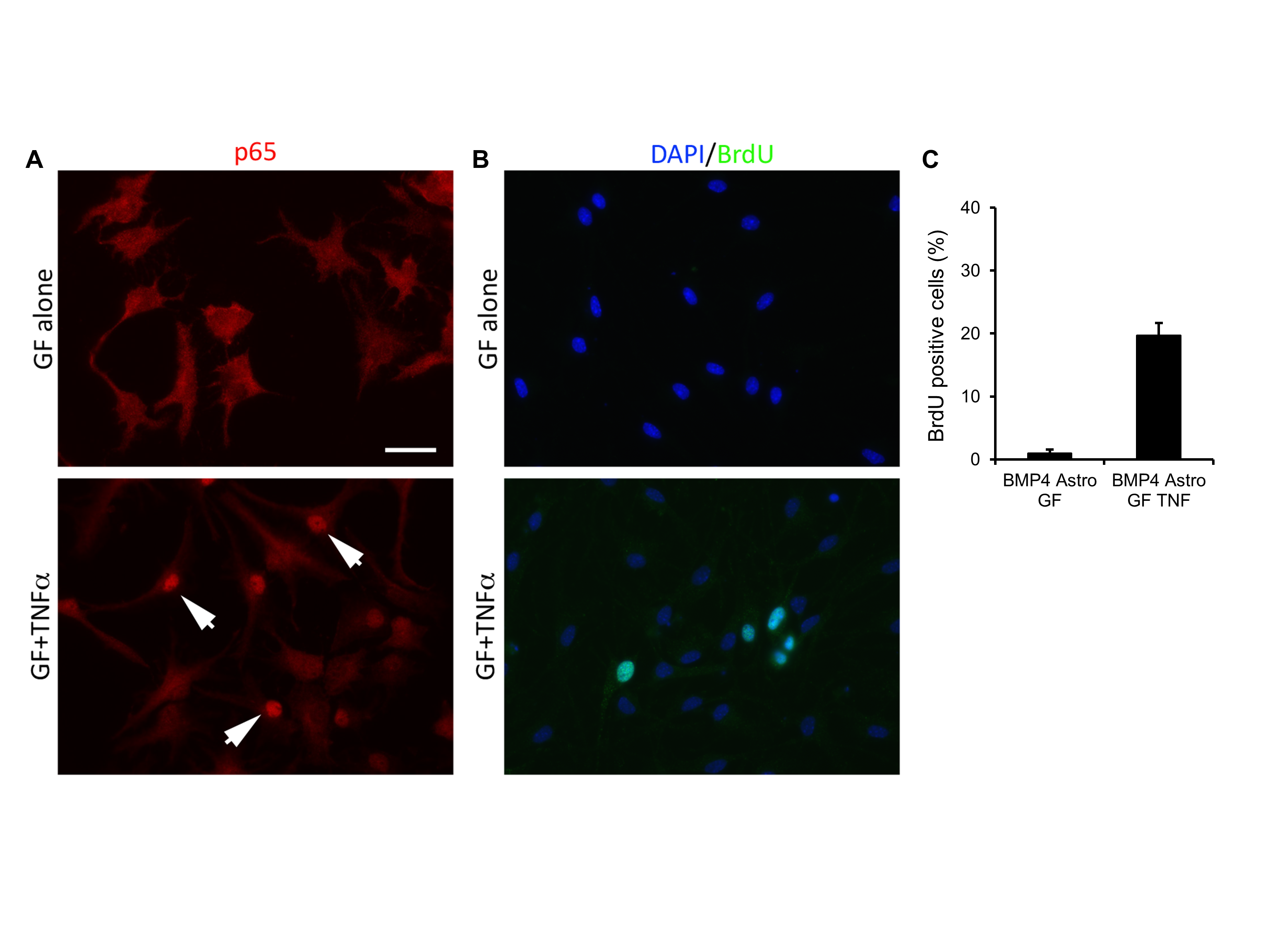

Supplement: Supplementary file 20 — High resolution (TIFF 1,560 kb) [file 12035_2015_9296_MOESM16_ESM.tif]

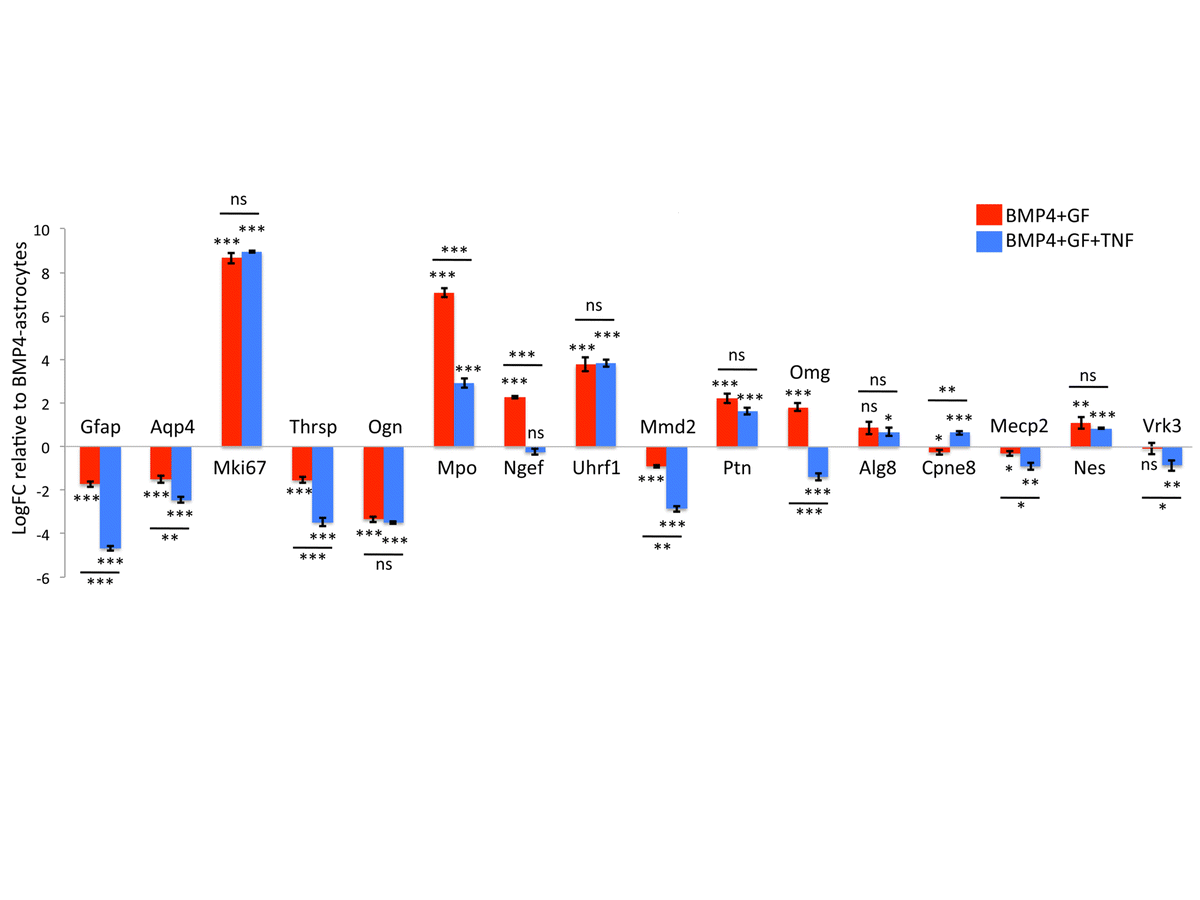

Supplement: Supplementary file 21 — (GIF 48 kb) [file 12035_2015_9296_Fig12_ESM.gif]

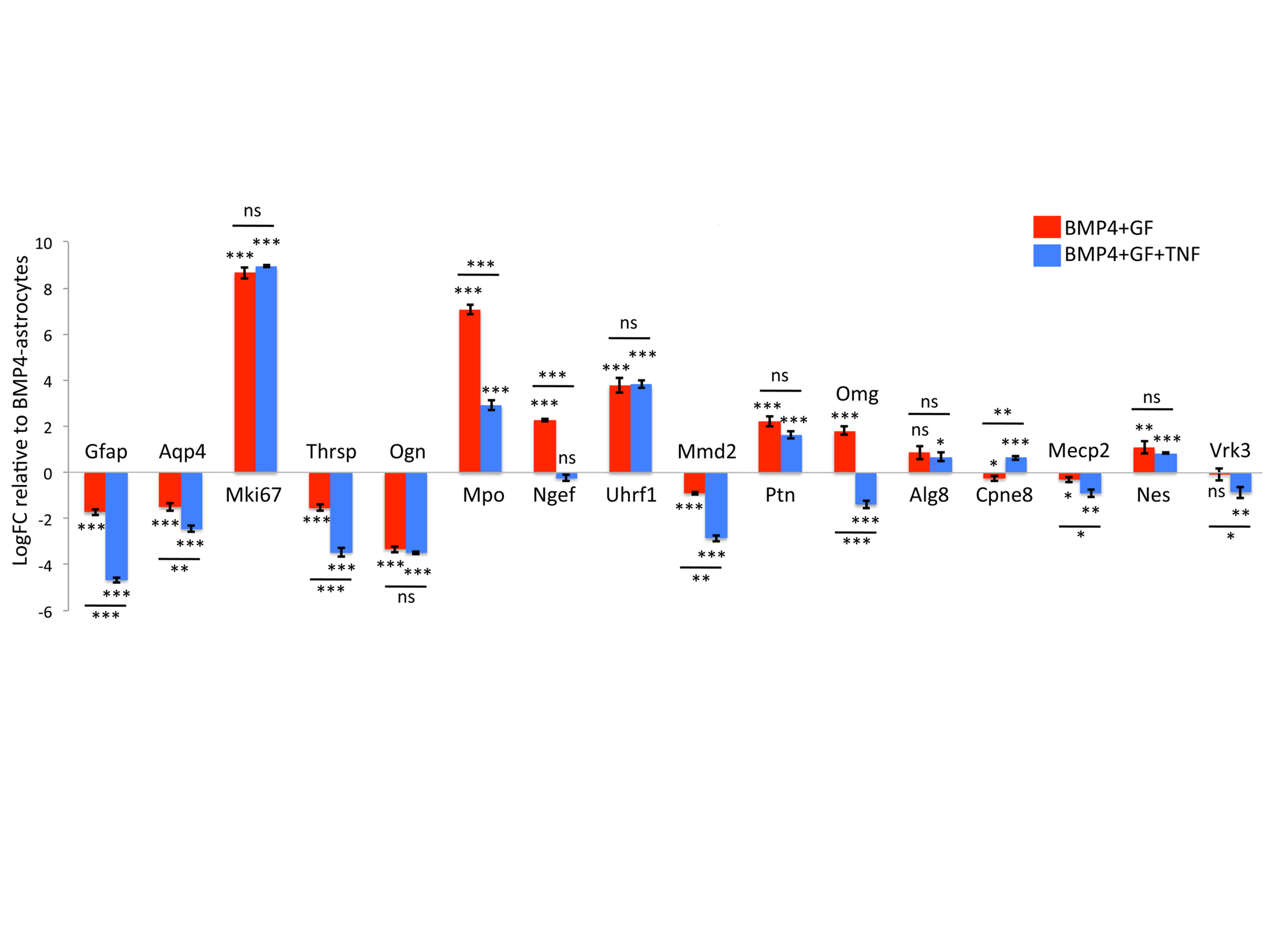

Supplement: Supplementary file 22 — High resolution (TIFF 505 kb) [file 12035_2015_9296_MOESM17_ESM.tif]

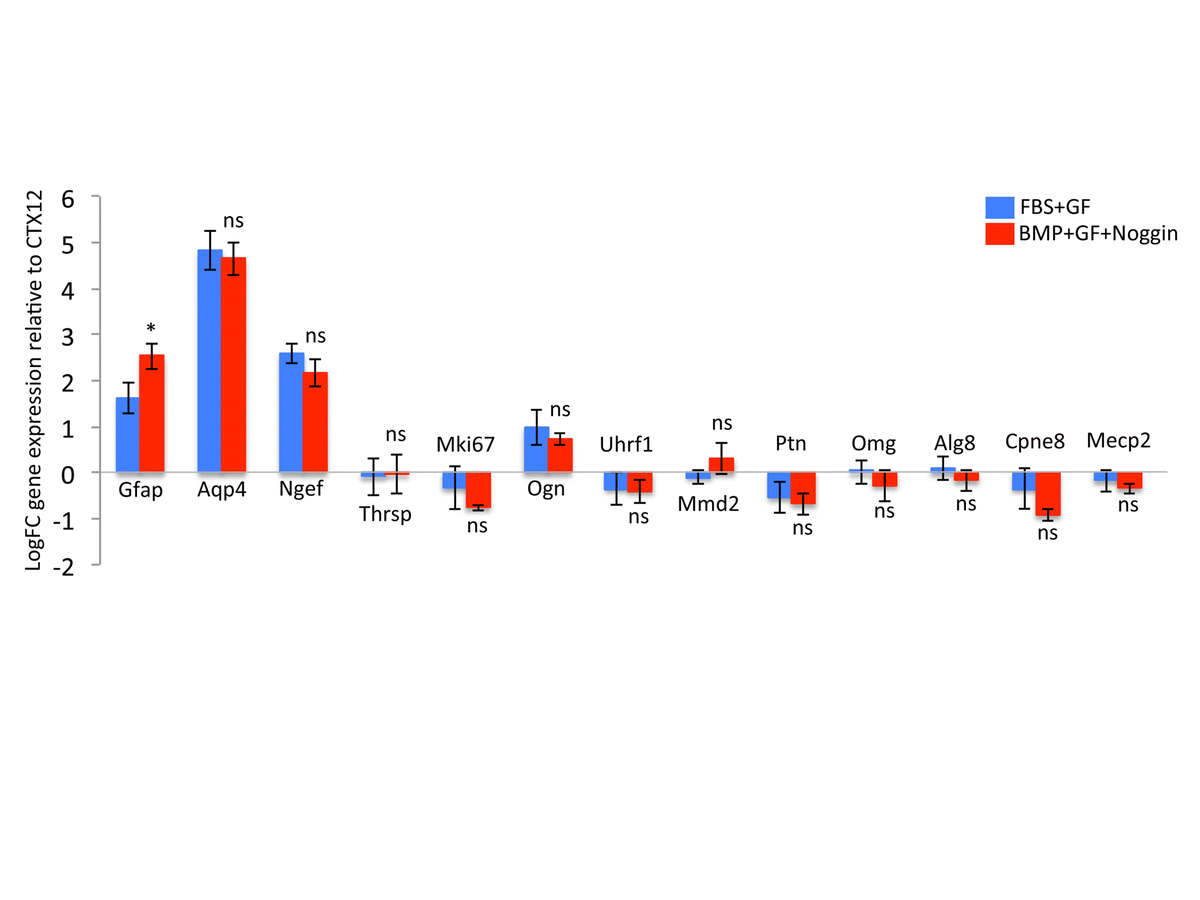

Supplement: Supplementary file 23 — (GIF 44 kb) [file 12035_2015_9296_Fig13_ESM.gif]

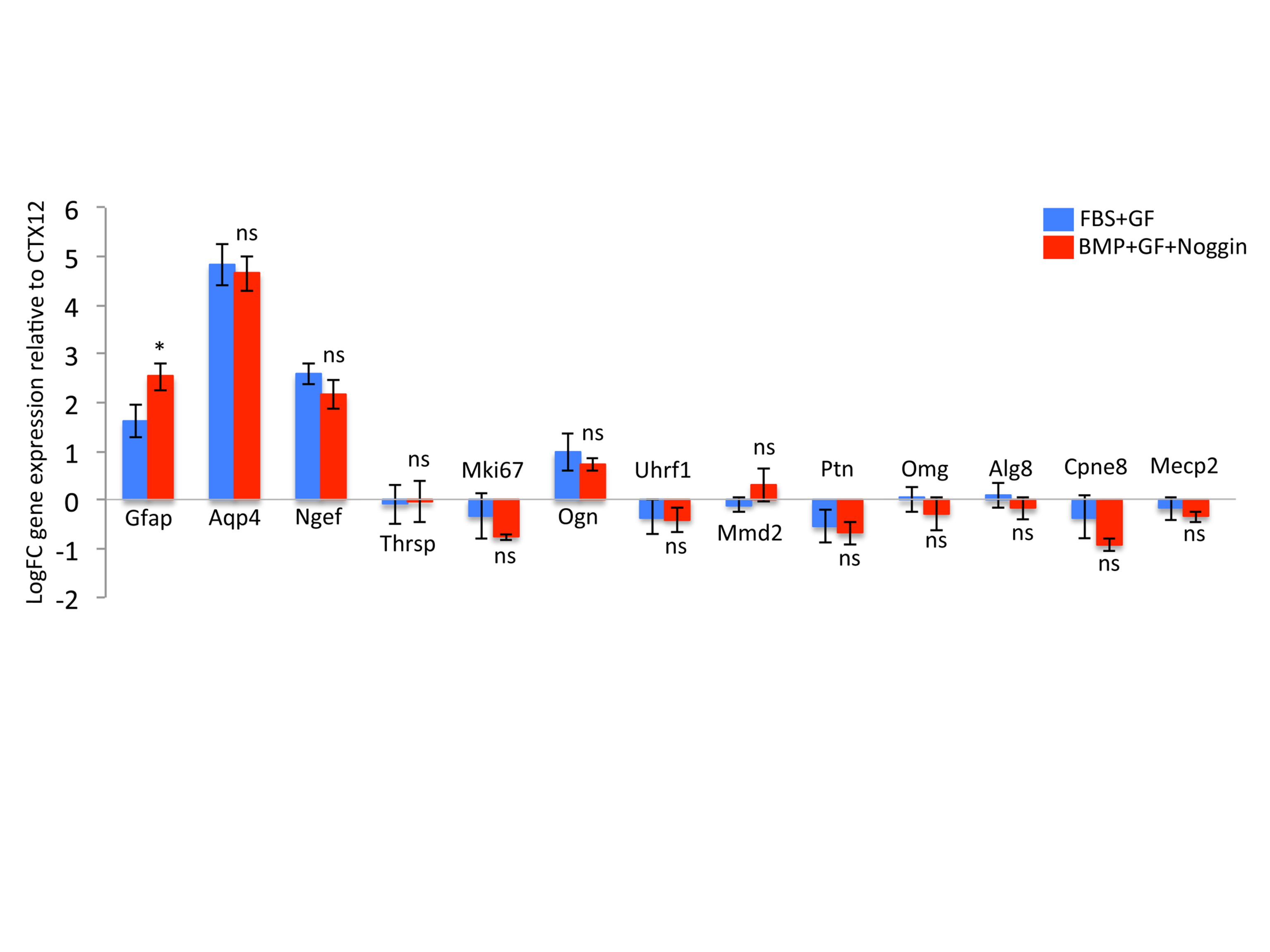

Supplement: Supplementary file 24 — High resolution (TIFF 454 kb) [file 12035_2015_9296_MOESM18_ESM.tif]

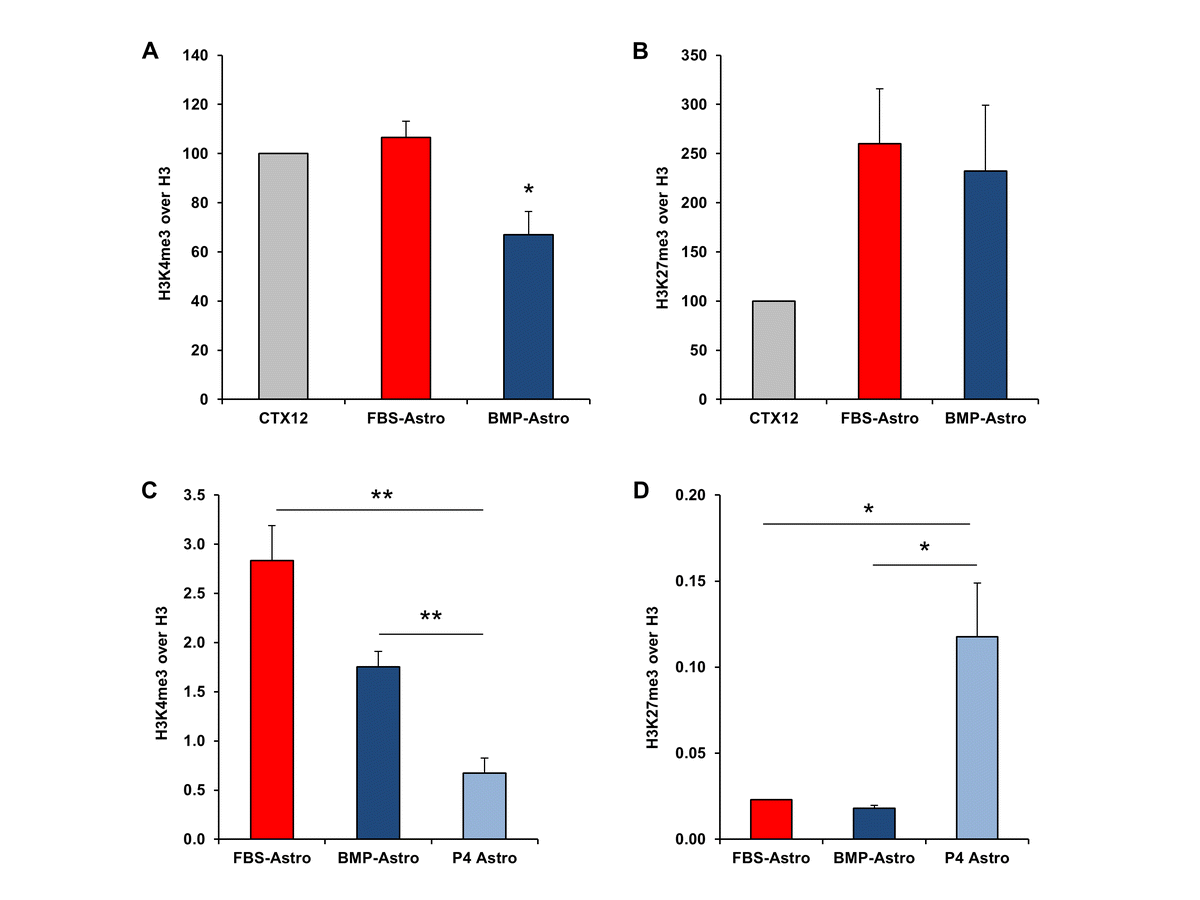

Supplement: Supplementary file 25 — (GIF 53 kb) [file 12035_2015_9296_Fig14_ESM.gif]

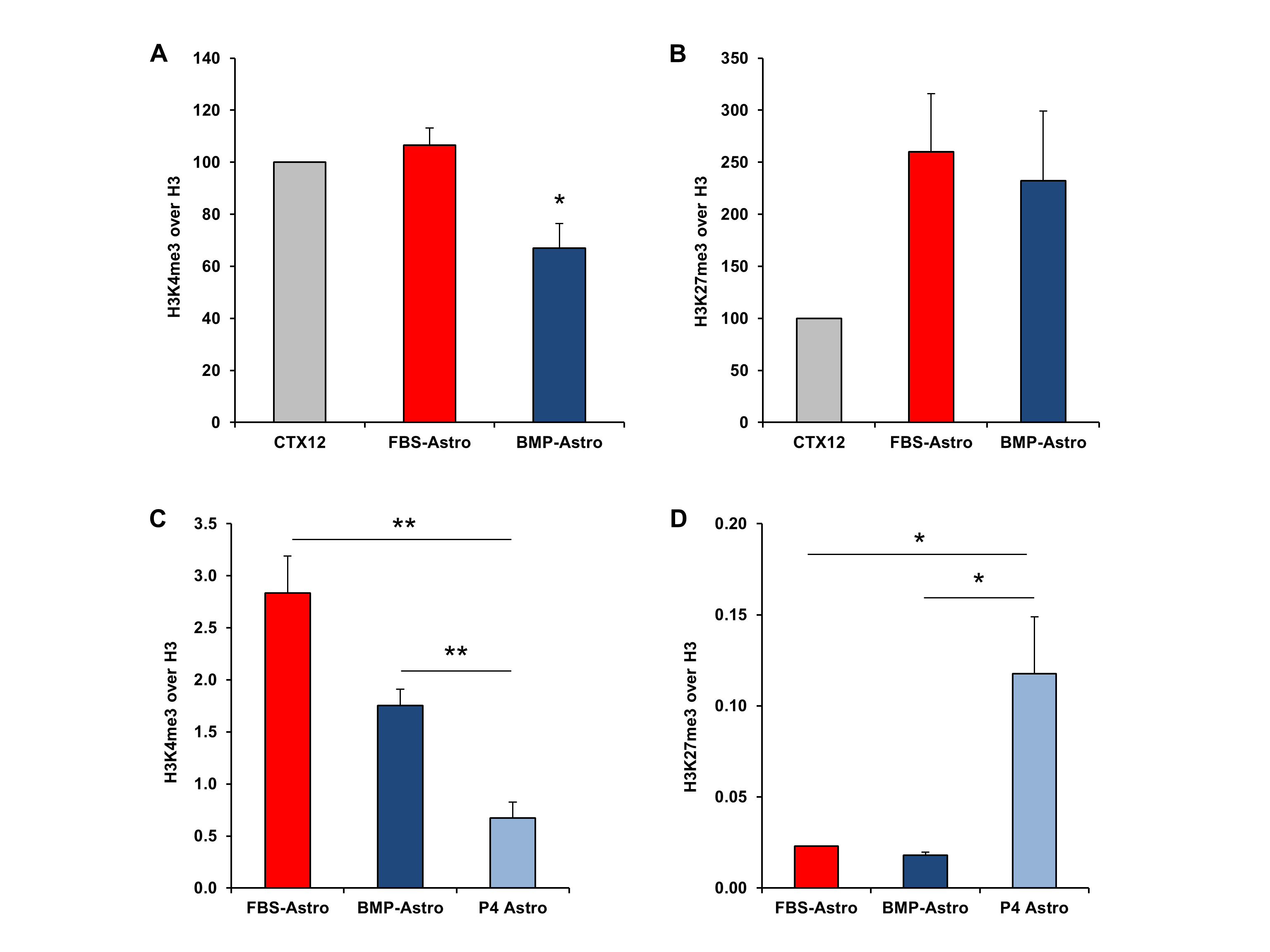

Supplement: Supplementary file 26 — High resolution (TIFF 189 kb) [file 12035_2015_9296_MOESM19_ESM.tif]

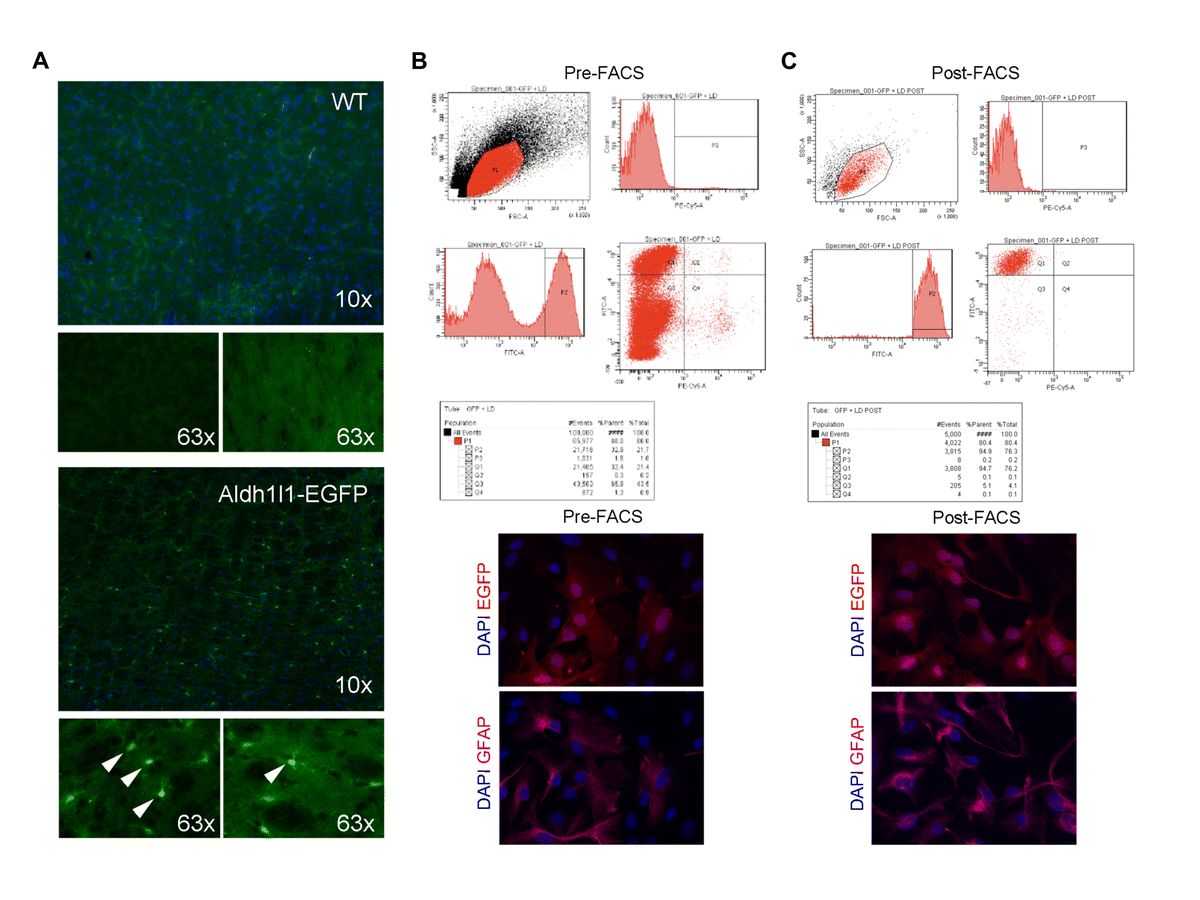

Supplement: Supplementary file 27 — (GIF 287 kb) [file 12035_2015_9296_Fig15_ESM.gif]

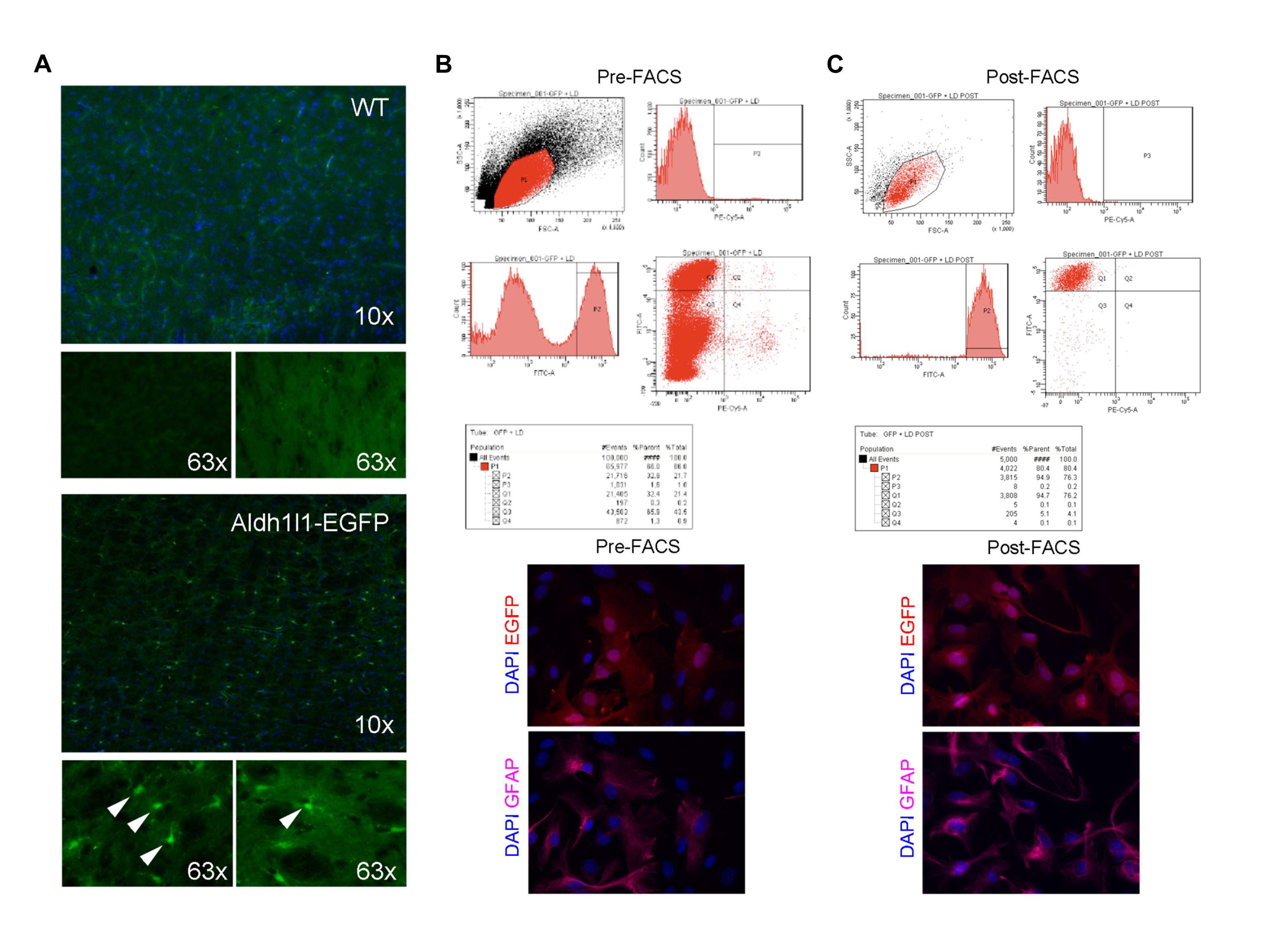

Supplement: Supplementary file 28 — High resolution (TIFF 4,037 kb) [file 12035_2015_9296_MOESM20_ESM.tif]

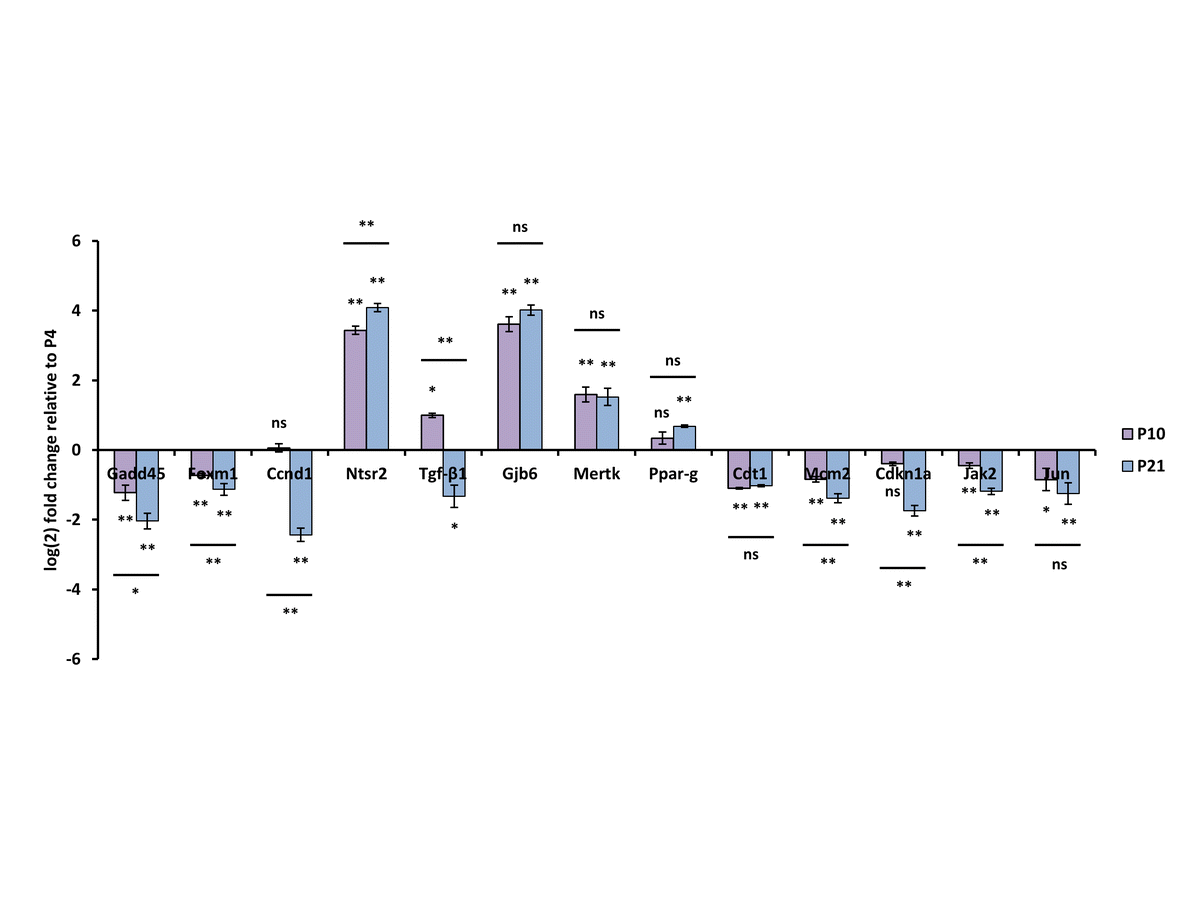

Supplement: Supplementary file 29 — (GIF 37 kb) [file 12035_2015_9296_Fig16_ESM.gif]

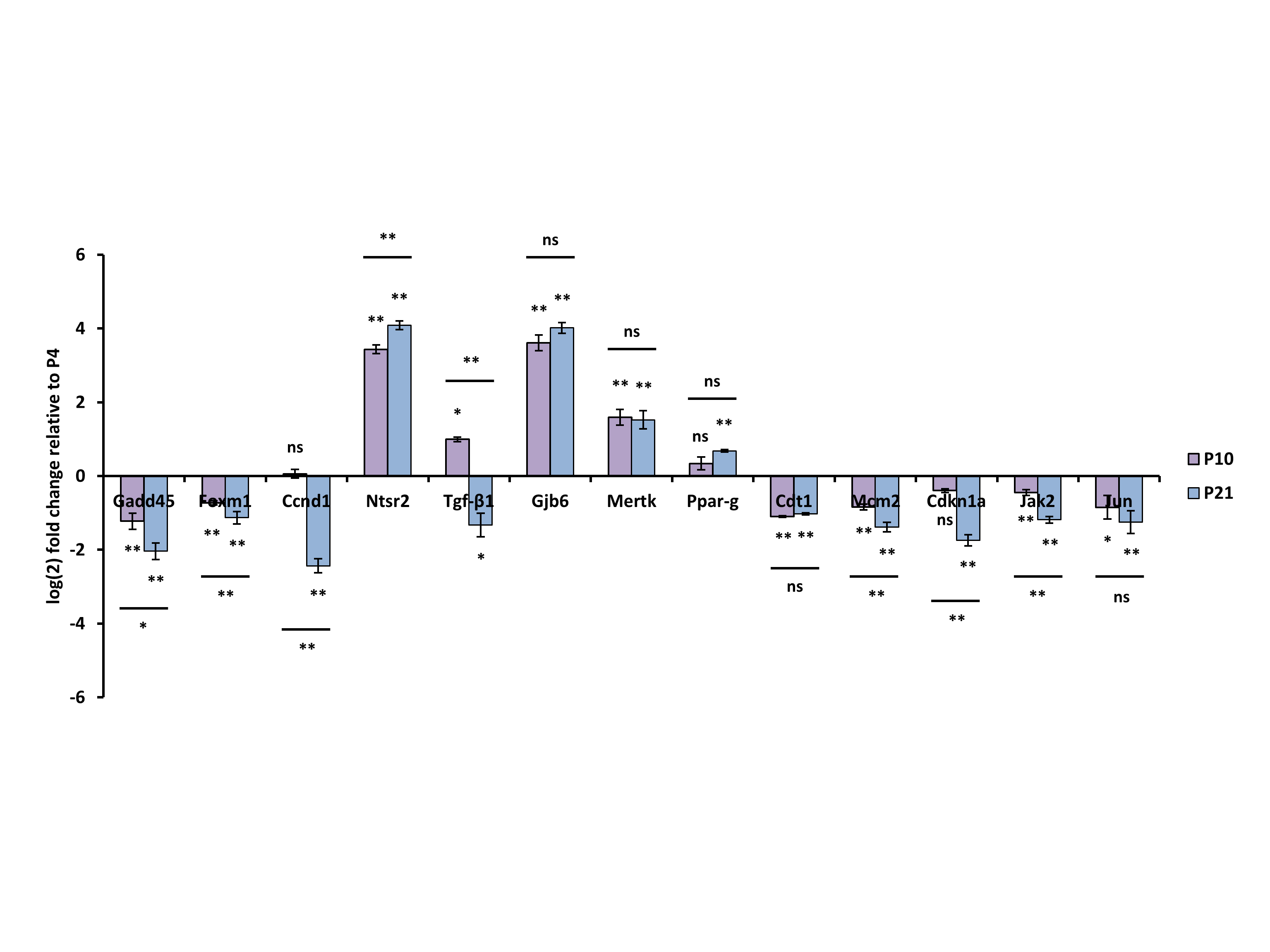

Supplement: Supplementary file 30 — High resolution (TIFF 136 kb) [file 12035_2015_9296_MOESM21_ESM.tif]

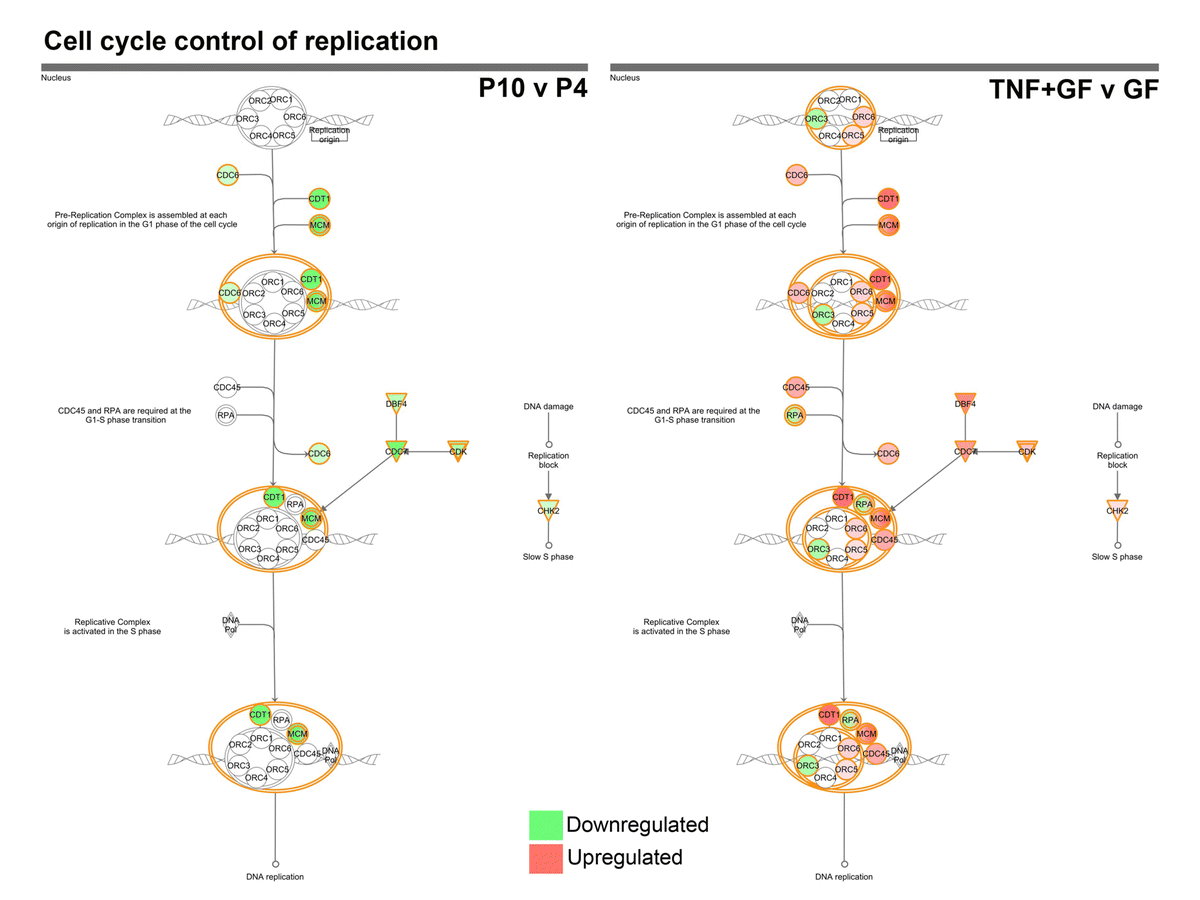

Supplement: Supplementary file 31 — (GIF 104 kb) [file 12035_2015_9296_Fig17_ESM.gif]

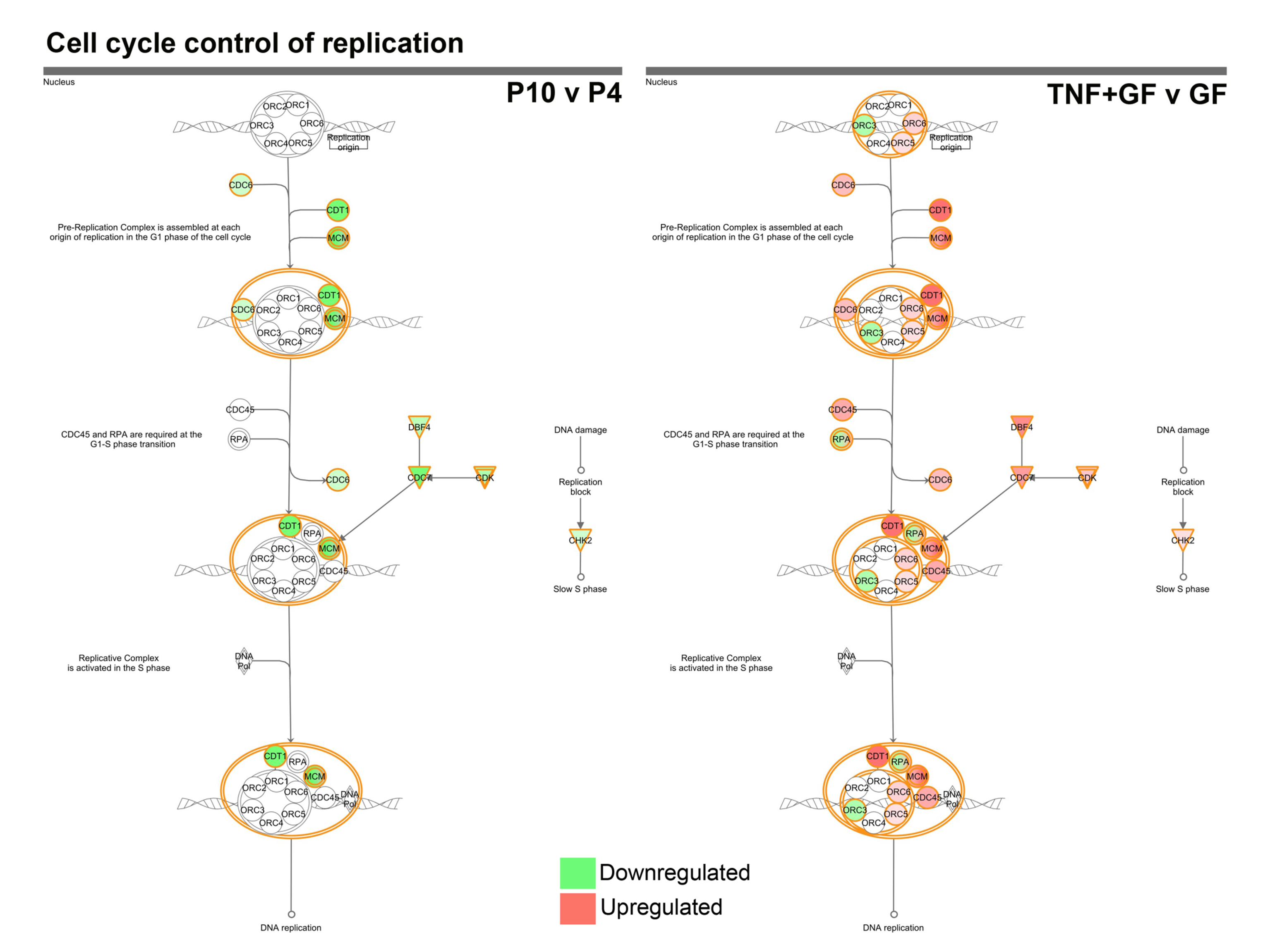

Supplement: Supplementary file 32 — High resolution (TIFF 1,790 kb) [file 12035_2015_9296_MOESM22_ESM.tif]

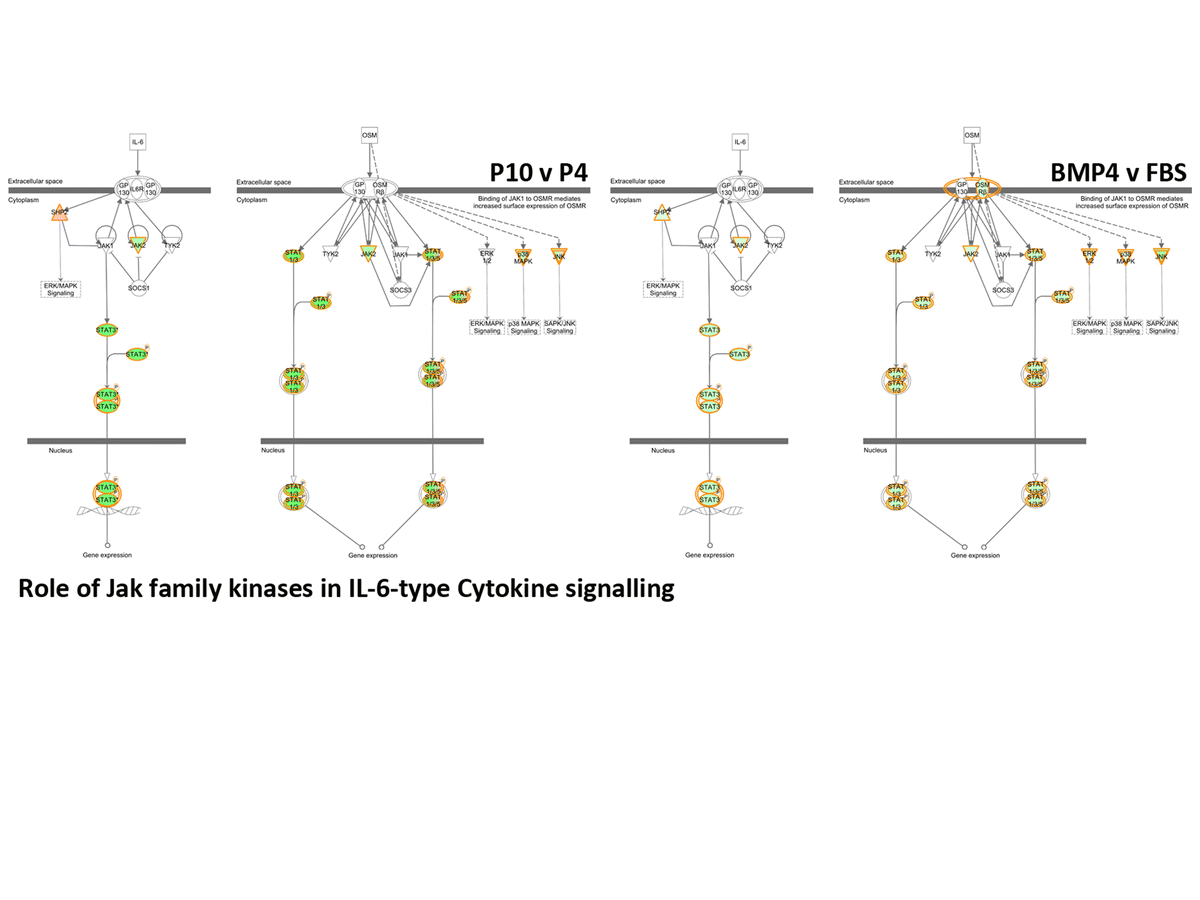

Supplement: Supplementary file 33 — (GIF 64 kb) [file 12035_2015_9296_Fig18_ESM.gif]

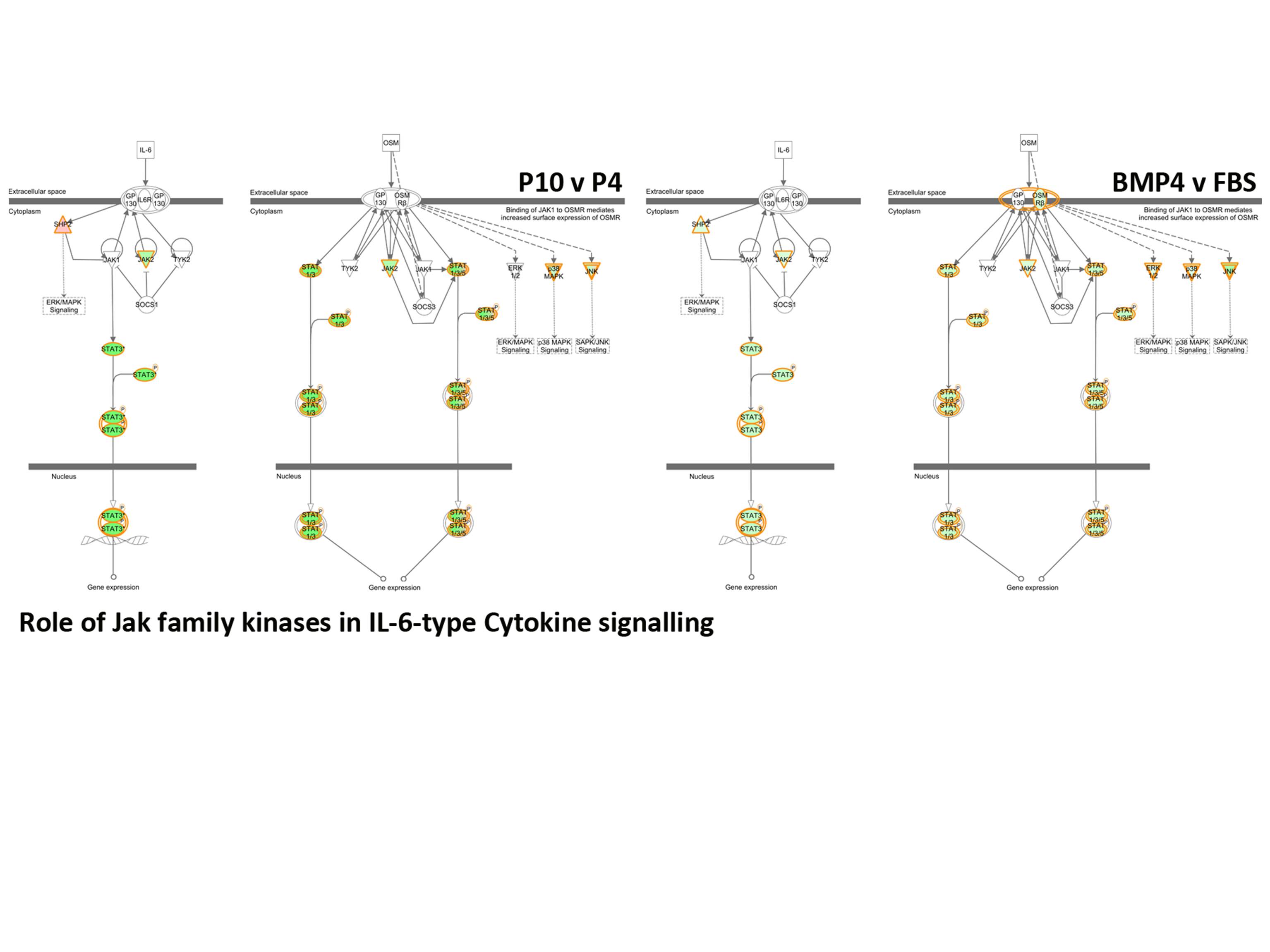

Supplement: Supplementary file 34 — High resolution (TIFF 948 kb) [file 12035_2015_9296_MOESM23_ESM.tif]
